# Interplay between NRF2 post-translational modifications and protein-protein interactions: Perspectives from emerging structural and functional evidence

**DOI:** 10.1016/j.abb.2026.110847

**Published:** 2026-08

**Authors:** Adem Ozleyen, Seda Savranoglu Kulabas, Miroslav Novak, Milena Cichoń, Cristina Matas De Las Heras, Tadashi Honda, Richard G. Doveston, Albena T. Dinkova-Kostova, Anna Grochot-Przeczek, Tugba Boyunegmez Tumer

**Affiliations:** a Health Institutes of Turkiye, Turkiye Biotechnology Institute, Ankara, 06270, Turkiye; b Department of Immunology, Faculty of Medicine, Çanakkale Onsekiz Mart University, Çanakkale, Turkiye; c Division of Cancer Research, School of Medicine, University of Dundee, Scotland, United Kingdom; d Department of Medical Biotechnology, Faculty of Biochemistry, Biophysics and Biotechnology, Jagiellonian University, Kraków, Poland; e Doctoral School of Exact and Natural Sciences, Jagiellonian University, Kraków, Poland; f Leicester Institute for Structural and Chemical Biology, University of Leicester, Leicester, LE1 7RH, United Kingdom; g School of Chemistry, University of Leicester, Leicester, LE1 7RH, United Kingdom; h Department of Chemistry and Institute of Chemical Biology & Drug Discovery, Stony Brook University, Stony Brook, NY, USA; i Department of Physiology, Pharmacology and Therapeutics, Johns Hopkins University School of Medicine, Baltimore, MD, USA; j Department of Medicine, Johns Hopkins University School of Medicine, Baltimore, MD, USA; k Department of Molecular Biology and Genetics, Faculty of Arts and Science, Canakkale Onsekiz Mart University, Canakkale, 17020, Turkiye

**Keywords:** NRF2, PPIs, KEAP1, PTM-Code, Regulatory mechanisms

## Abstract

Nuclear factor erythroid 2-related factor 2 (NRF2), a redox-sensitive transcription factor, is a master regulator of cellular adaptation to diverse types of stressors. Under basal conditions, the regulation of NRF2 is governed by Kelch-like ECH-associated protein 1 (KEAP1), an adaptor subunit of the CUL3-based E3 ubiquitin ligase, which promotes the ubiquitination and subsequent degradation of NRF2. However, when electrophilic or oxidative stressors alter the conformation of the KEAP1–NRF2 complex, KEAP1 loses its regulatory control over newly synthesized NRF2, leading to its accumulation and nuclear translocation, where it exerts transcriptional activity. NRF2 stability and activity are also shaped by a broader spectrum of protein–protein interactions (PPIs), including recently emerging regulators such as peptidyl prolyl isomerase (PIN1). Significantly, many of these dynamic PPI networks are regulated by post-translational modifications (PTMs), which, in turn, can be governed by these PPIs. While major PTMs such as phosphorylation and ubiquitination constitute the central regulatory processes, atypical or less-characterized modifications, including SUMOylation and O-GlcNAcylation, are gaining increasing attention for their tissue and condition-specific roles. This review compiles the latest structural and functional evidence on well-known as well as understudied PTMs and PPIs of NRF2, emphasizing the dynamic interplay between these regulatory mechanisms in shaping NRF2 signaling under physiological and stress conditions.

## Highlights

• Compartmentalized PTMs and partner crosstalk collectively orchestrate NRF2 activity.
• Phosphorylation events modulate NRF2 binding to KEAP1, β-TrCP, PIN1, and others.
• Noncanonical PTMs and condition-specific partners dynamically reshape NRF2 function.
• The regulatory relevance and downstream impact of NRF2 S40 phosphorylation remain open.
• Modulating NRF2 interaction networks offers selective therapeutic opportunities.

## Graphical abstract

**Figure:**
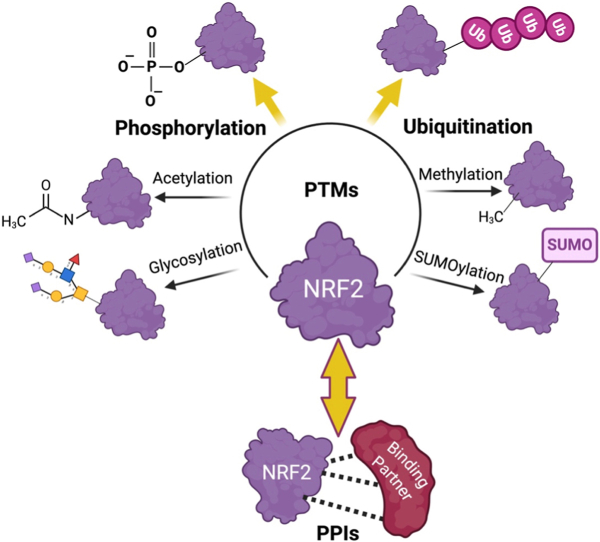

## 1 Introduction

Transcription factors (TFs) are among the most dynamic subsets of the cellular proteome. Their activity, stability, and subcellular localization are tightly regulated by a complex network of post-translational modifications (PTMs) and protein-protein interactions (PPIs), which together precisely adjust prompt transcriptional responses to environmental and intracellular triggers. Nuclear factor erythroid 2-related factor 2 (NRF2), the major TF mediating a rapid and robust adaptive response to maintain redox homeostasis against cellular stress, is a prime example of such a dynamic nature, governed by its intricate crosstalk between post-translational control mechanisms and an extensive interactome [1]. The landscape of NRF2 research has undergone significant evolution since its initial conceptualization nearly three decades ago [2]. Over this time, extensive investigations have revealed the voluminous roles of NRF2 in regulating transcription, antioxidative responses, autophagy, apoptosis, and cell cycle progression, along with the molecular mechanisms that govern these processes [3]. Initial research primarily focused on the Kelch-like ECH-associated protein 1 (KEAP1)-dependent regulation of NRF2, which is recognized as the primary mechanism controlling its activity. However, subsequent research has identified a diverse array of conditional binding partners that modulate NRF2 activity, further highlighting its regulatory complexity. These partners contribute to the stabilization and activation or inactivation of NRF2 under various physiological and pathological conditions, thereby broadening its functional repertoire.

Under basal cellular conditions, the intracellular half-life of NRF2 is tightly controlled by the cytosolic protein KEAP1. KEAP1 binds NRF2 in the cytoplasm, sequestering it from the nucleus and promoting its ubiquitination and subsequent proteasomal degradation, a process that takes less than 30 min [4]. However, further studies have revealed alternative, predominantly stress-dependent regulatory mechanisms that modulate NRF2 activity, either enhancing or suppressing its function. These pathways involve a diverse set of binding partners, including corepressors such as the well-known β-transducin repeat-containing protein (β-TrCP) and the relatively less-studied glucocorticoid receptor (GR) and retinoid X receptor α (RXRα), as well as coactivators such as CREB-binding Protein (CBP), mediator complex subunit 16 (MED16), receptor-associated coactivator 3 (RAC3), and the recently identified peptidyl-prolyl *cis*-trans isomerase NIMA-interacting 1 (PIN1) [5]. Although the intricate proteomic landscape of these interactions remains incompletely understood, PTMs play a pivotal role in regulating NRF2 through PPI-dependent mechanisms beyond KEAP1. The major PTMs that regulate NRF2 stability and activity are ubiquitination, predominantly driven by the KEAP1–CUL3 axis, and phosphorylation, mediated by multiple stress-responsive kinases. Moreover, acetylation, SUMOylation, methylation, and O-GlcNAcylation have been documented as modulators of NRF2. Nevertheless, these modifications are regarded as being less well-characterized and more condition-dependent compared to ubiquitination and phosphorylation [6].

Initially, PTMs were viewed primarily as terminal events that dictated the fate of downstream signaling pathways. However, it is now increasingly recognized that these modifications, and the emerging concept of a PTM code, have broader functional implications. In particular, PTMs can modulate PPIs, thereby extending their influence beyond individual modification events and reshaping entire signaling cascades. To support research in this area, the PTMcode database (https://ptmcode.embl.de/index.cgi) provides a comprehensive dataset of experimentally validated and computationally predicted PTMs, along with their functional associations, encompassing more than 100,000 proteins [7]. Specifically for NRF2, PTMcode currently annotates 43 PTMs, including 36 experimentally validated modifications and 7 additional predicted sites. These PTMs are linked to 192 distinct biological functions (last accessed on 17 November 2025). Compared with other PTM databases, this platform stands out for its user-friendly, straightforward interface. Nevertheless, the resource would benefit from modernizing its web implementation to further enhance accessibility and usability.

**Fig. 1:**
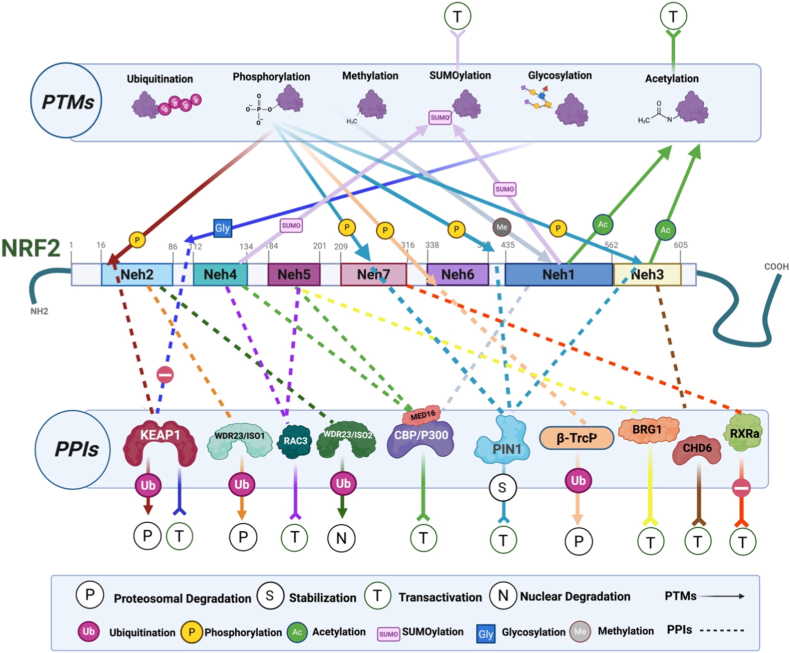
Schematic representation of PTM-mediated PPIs and PPI-mediated PTMs in NRF2 regulation. Phosphorylation and ubiquitination are two major, well-characterized functional PTMs that promote NRF2's association with its binding partners. While PTMs dictate PPIs, PPIs can in turn induce or modulate PTMs on NRF2, illustrating a bidirectional regulatory relationship.

Understanding how NRF2 PTMs influence its binding affinity for partner proteins and, conversely, how PPIs shape the pattern of NRF2 modifications could yield critical insights into its regulatory dynamics. This bidirectional relationship between PTMs and PPIs, rather than acting as isolated events, creates a regulatory crosstalk in which one modification often dictates the occurrence of the other (Fig. 1). For instance, phosphorylation of the Neh6 domain by glycogen synthase kinase-3 beta (GSK-3β) generates a phosphodegron motif that promotes the recruitment of the β-TrCP E3 ligase complex, thereby coupling a PTM event to a subsequent protein–protein interaction leading to degradation [8]. Conversely, the canonical interaction between the Neh2 domain and KEAP1 precedes a cascade of ubiquitination events that determine NRF2 turnover under basal conditions [9]. Such interdependence highlights that NRF2 regulation cannot be viewed merely as a sequence of linear modifications or interactions but rather as a dynamic and self-reinforcing network, where PTMs and PPIs continuously influence one another (Fig. 1).

Excellent reviews have thoroughly detailed the molecular mechanisms governing NRF2 regulation (see Refs. [3,10] for comprehensive reviews). Here, we propose that NRF2 regulation can be broadly categorized into two interconnected, bidirectional levels: PTM-mediated PPIs and PPI-mediated PTMs. Despite substantial progress in the NRF2 field, many aspects of this bidirectional regulation remain incompletely understood. In this review, we also highlight these unresolved regulations and provide a comprehensive and updated perspective on the dynamic interplay between PTMs and PPIs in NRF2 signaling.

## 2 PTMs of NRF2 modulating its partner protein interactions

**Fig. 2:**
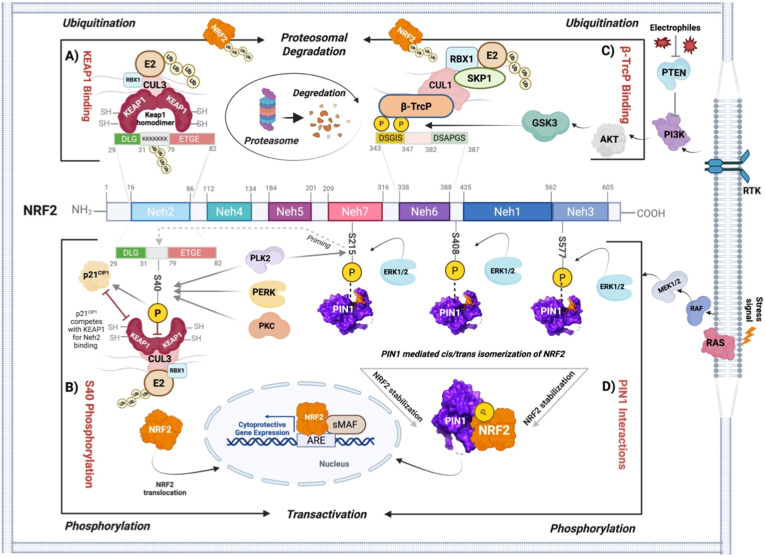
Representation of how PTMs modulate NRF2's PPIs. **(A)** Under canonical regulation, NRF2 is maintained at low basal levels through KEAP1-mediated ubiquitination and subsequent proteasomal degradation. **(B)** However, PKC-dependent phosphorylation of NRF2 at S40 has been shown to impair KEAP1 binding, thereby attenuating its ubiquitination and degradation. **(C)** In contrast to the KEAP1 pathway, NRF2 phosphorylation is required to facilitate its interaction with β-TrCP, triggering an alternative ubiquitination pathway that leads to NRF2 degradation. **(D)** More recently, a non-canonical NRF2-interacting partner, PIN1, has been identified, which requires NRF2 phosphorylation as a priming step for binding. This interaction promotes NRF2 stabilization and enhances its transcriptional activity.

This section summarizes the PTMs that modulate NRF2's subsequent interactions with its binding partners. Among various PTMs, phosphorylation stands out as the most extensively characterized and mechanistically significant molecular switch that determines the strength, specificity, and timing of PPIs. Phosphorylation events within distinct NRF2 domains, particularly those catalyzed by kinases such as protein kinase C (PKC), protein kinase RNA-like endoplasmic reticulum kinase (PERK), polo-like kinase 2 (PLK2), GSK-3β and mitogen-activated protein kinases (MAPKs) can either promote or prevent the recruitment of E3 ligases, adaptor proteins, enzymes, and coactivators, thereby determining stability and transcriptional efficacy of NRF2 [10] (Fig. 2). Understanding how phosphorylation orchestrates these PPIs provides critical insight into the dynamic control of NRF2 signaling under physiological and stress conditions.

### 2.1 Phosphorylation of NRF2 within Neh2 domain and its modulatory role in KEAP1 interaction

#### 2.1.1 KEAP1-NRF2 interaction

The first discovered repressor of NRF2 was KEAP1 [9], a substrate adaptor for a CUL3-based ubiquitin ligase, which facilitates the proteasomal degradation of NRF2 under basal circumstances; the inhibition of this process following the detection of oxidants or electrophiles, which modify reactive cysteines of KEAP1, allows NRF2 stabilization and activation to occur rapidly [11]. Seven NRF2-ECH (Neh) domains have been recognized within the NRF2 protein (Fig. 2). Six (Neh1-6) were identified as highly conserved regions between mammalian and avian NRF2, the latter then known as ECH [9], while Neh7 was added later based on its function in interacting with RXRα [12]. The Neh2 domain, found on the N terminus of NRF2 (residues 16-86), is responsible for the KEAP1-dependent negative regulation of NRF2-driven gene expression [9].

KEAP1 contains three named domains: the broad complex-tramtrack-bric-a-brac (BTB) domain, the intervening region (IVR), and the Kelch domain; the last one interacts with NRF2. This layout is standard among all substrate adaptors of the Kelch-like (KLHL) protein family; KEAP1 is also known as KLHL19 [13]. However, neither the Kelch domain arginines responsible for NRF2 binding nor the electrophile-reactive cysteines are found in KLHL proteins other than KEAP1 [14,15].

The Kelch domain of KEAP1 recognizes two sites in the Neh2 domain of NRF2: DLG (residues 29-31) and ETGE (residues 79-82), with a greater than 50-fold higher affinity for the latter [16] (Fig. 2A). Since the sites bind to overlapping surfaces of the Kelch domain, one KEAP1 molecule can't interact with both Neh2 sites. However, the BTB domain of KEAP1 facilitates homodimerization, which is essential for the ubiquitination of NRF2 [17]. Ubiquitination thus occurs when the DLG and ETGE sites are bound by the two KEAP1 molecules of the dimer, which stabilizes the α-helix central to the Neh2 domain, allowing ubiquitin conjugation to its lysines [18]. This is known as the ‘closed’ conformation; in the ‘open’ conformation, where only the higher-affinity ETGE site is bound, the flexibility of the positioning of the α-helix prevents its productive ubiquitination [19].

The high affinity between the Neh2 and Kelch domains, in combination with the ease of NRF2 polyubiquitination in the closed conformation, results in a sub-30-min half-life of the NRF2 protein, as has been observed in a range of mammalian cell lines [8,[20], [21], [22], [23], [24], [25]]. In some cancers, most commonly lung cancers, however, the negative regulation of NRF2 activity by KEAP1 is absent due to loss-of-function mutations in KEAP1 or epigenetic DNA modifications that suppress *KEAP1* expression [26,27]. Alternatively, NRF2 can be mutated partly, and while cancer-associated KEAP1 variants are found throughout the protein, NRF2 variants primarily affect the DLG and ETGE motifs [28], highlighting the oncosuppressive role of KEAP1. Additionally, transcript variants lacking exon 2 and exons 2 + 3 of *NFE2L2*, resulting in the inability of NRF2 to bind KEAP1, have also been reported [29].

#### 2.1.2 Mechanism of phosphorylation and binding

Alongside the seven redundantly ubiquitinated lysines (described in detail in section 3.1.1), one other PTM has been recognized in the Neh2 domain: S40 phosphorylation (Fig. 2B). It was first shown in vitro that protein kinase C (PKC) can phosphorylate wild-type NRF2 but not its S40A mutant [30]. This mutation did not impair KEAP1, MAFK, or DNA binding; however, upon co-incubation with PKC, KEAP1 disappeared from the immunoprecipitate of wild-type NRF2 but not S40A NRF2. As the PKC inhibitor staurosporine prevented the apparent dissociation, it was postulated that, by phosphorylating it on S40, PKC promotes the release of NRF2 from KEAP1 [30]. The involvement of PKC in NRF2 activation was further highlighted in HepG2 cells, where inhibitors of PKC, but not MEK/MAPK, PI3K, or PTKs, suppressed the expression of NAD(P)H:quinone oxidoreductase (*NQO1*), a classical target gene of NRF2 [31]. Furthermore, in Hepa-1 cells treated with *tert*-butylhydroquinone (tBHQ), staurosporine prevented the nuclear accumulation of NRF2, and in the absence of the PKC inhibitor, the NRF2 translocating to the nucleus displayed increased phosphorylation specifically on serine [31], suggesting PKC-mediated NRF2 phosphorylation on S40 was required for its activation in the presence of electrophilic stressors.

At a similar time, PERK was identified as an NRF2-binding protein using a yeast two-hybrid screen [32]. ER stressors tunicamycin and thapsigargin induced the phosphorylation, nuclear translocation, and activation of NRF2 in NIH 3T3 cells; this did not occur in cells lacking PERK or expressing its dominant negative variant, while wild-type PERK overexpression resulted in nuclear NRF2 even in the absence of ER stress [32]. *In vitro*, PERK promoted the separation of the NRF2:KEAP1 complex, and NRF2 pre-incubated with PERK was refractory to KEAP1 binding, while its interaction with MAFG and DNA remained unaffected [32]. As *PERK* knockdown lowered both NQO1 and NRF2 pS40 levels in ARPE-19 cells [33], an argument can be made for PERK's participation in NRF2 phosphorylation on S40.

Additionally, PLK2, a tumour-suppressive kinase positively regulated by p53 whose downregulation is associated with chemoresistance [34], has been observed to phosphorylate NRF2 on S40 and S215 in vitro [35]. In HepG2 cells, treatment with the anthracycline antibiotic doxorubicin triggered an increase in NRF2 protein levels and S40 phosphorylation; these changes were suppressed by the knockdown of *PLK2,* which is usually upregulated in response to doxorubicin exposure [35]. The phosphomimetic S40E mutant of NRF2 was found to promote the expression of heme oxygenase 1 (*HMOX1*), another NRF2 target gene, and interact less with KEAP1 but more with p21^CIP1^ [35]. The interaction of the classical p53 target p21^CIP1^ and NRF2 was described 10 years earlier, when it was found that p21^CIP1^ competes with KEAP1 for Neh2 binding – and while the affinity of p21^CIP1^ is not high enough to displace KEAP1, NRF2 half-life and activity are suppressed in p21^CIP1^–KO conditions, both basally and following treatment with the electrophilic NRF2 activators tBHQ or sulforaphane (SFN) [25]. While overexpressing the S215E NRF2 mutant did not affect its activity *per se*, the PLK2 association and S40 phosphorylation were affected positively, suggesting that S215 phosphorylation may prime NRF2 for the subsequent phosphorylation on S40 [35].

The discoveries mentioned above establish a consensus function for NRF2 S40 phosphorylation, in which PKC, PERK, or PLK2, activated by different stimuli, phosphorylate this residue to trigger its release from KEAP1, interaction with p21^CIP1^, nuclear translocation, and transcriptional activity (Fig. 2B).

#### 2.1.3 AKT or p38 through unknown intermediaries?

In a recent study (still in a pre-print format at the time of writing), Manfreda et al. studied the chemotherapy-sensitive and -resistant versions of three different medulloblastoma cell lines: HD-MB03, DAOY, and HuTuP33 [36]. In all three cases, the resistant cell lines exhibited higher levels of NRF2 activity (as evidenced by NQO1 levels) and S40 phosphorylation (while total protein levels remained unchanged). As both NQO1 and NRF2 pS40 levels were lowered upon treatment with the PKC inhibitor enzastaurin [36], the effects were attributed to this kinase family, similarly to the findings described earlier. However, as has been shown multiple times in various cell lines, alongside PKC, enzastaurin also inhibits AKT [[37], [38], [39]]. This agrees with PKC's assigned position upstream of AKT [40,41].

That AKT positively affects NRF2 activity has been well established [42]. This effect, which can even be observed in KEAP1-mutant A549 cells [43], primarily relates to GSK3 inhibition and will be discussed later in section 2.2.2. But in a study using the triple-negative breast cancer cell line MDA-MB-231, NRF2 levels have been found to correlate inversely with those of the microRNA miR-29b-1-5p [44]. Overexpressing this microRNA depleted the levels of S473-phosphorylated AKT and S40-phosphorylated NRF2, without affecting the total levels of either protein. NRF2, however, was found to have migrated out of the nucleus, and the loss of its activity was consistent with a reduction in the NQO1 protein levels. MK-2206, an AKT inhibitor, affected both phosphoproteins the same way [44], suggesting that, independently of its role in NRF2 stabilization, AKT may also support its phosphorylation on S40. As the AKT consensus motif has been determined to require arginines at the −5 and −3 positions, and a hydrophobic residue at +1 [45], and the respective sequence surrounding the human NRF2 S40 is E-x-F-x-x-pS-Q, it is unlikely that this phosphorylation is carried out by AKT itself. PKC, as mentioned earlier, functions upstream of AKT, and, to our knowledge, no link between AKT and PLK2 activity has been established. AKT has been reported to inhibit PERK by phosphorylating it at T799 [46], although at the same time another group observed a possible non-canonical activation of PERK with the AKT inhibitor, Akt-IV [47].

In the search for other agents affecting NRF2 phosphorylation and localization but not overall levels, 3,4,5-trihydroxycinnamic acid (THCA) stands out: it has been shown to increase concentration-dependently NRF2 phosphorylation and translocation to the nucleus. NQO1 and HO-1 levels increased upon treatment with THCA, demonstrating NRF2 activation, while the activatory phosphorylations of AKT, ERK, JNK, NF-κB, IκBα were suppressed [48,49]. The exception was p38, which was activated by THCA, and pre-treatment with the p38 inhibitor SB203580 prevented its effect on NRF2 phosphorylation and activity [48]. The link between p38 and NRF2 activity has also been made in glioma cells after temozolomide treatment, however, as the total NRF2 protein levels increased with contributions from p38 in this case [50], the underlying signalling event(s) was likely different. Similarly to AKT, p38 is unlikely to phosphorylate NRF2's S40 itself, since it is a proline-directed kinase [51], but p38 has been implicated to regulate AKT positively [52], hinting at the possibility of a common pathway. The final kinase in this pathway – the one phosphorylating NRF2 directly – has not been validated yet, though. Even if it is the non-canonical PERK, a direct evidence is still needed.

#### 2.1.4 Revisiting the role of S40 phosphorylation

**Fig. 3:**
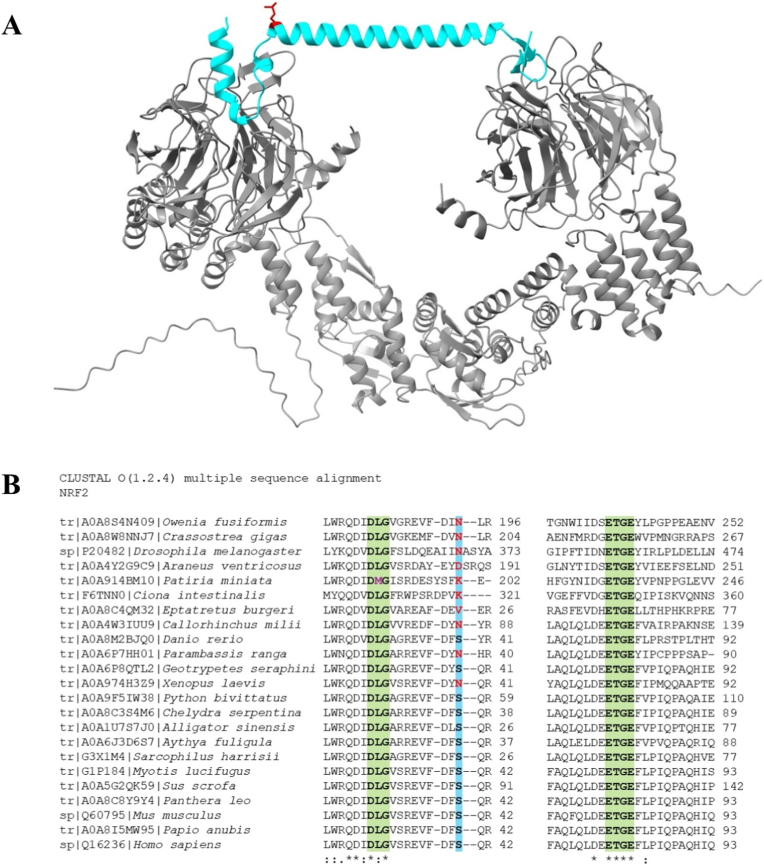
The predicted KEAP1-Neh2 pS40 complex. (**A)** This model was generated in AlphaFold 3 using the Q14145 (human KEAP1; gray) and Q16236 (human NRF2, residues 16-86; cyan) sequences, NRF2 phosphorylated on S40 (red). Analysis was performed using ChimeraX 1.9. (**B)** S40 is only conserved among amniote NRF2. Protein sequences were obtained from UniProt (accession numbers shown alongside species), sequence alignment was performed using Clustal Omega. S40 is highlighted in cyan (variant residues are red), DLG and ETGE motifs in green (the echinoderm variant is purple). (For interpretation of the references to colour in this figure legend, the reader is referred to the Web version of this article.)

Phosphorylation of NRF2 at S40 was initially thought to be essential for its activation under electrophilic stress; however, emerging evidence has put this narrative into question. Zhang and Hannink reported that the response of the S40A mutant to NRF2-activating molecules is no less compared to the wild-type protein [53]. Using electrophoretic mobility shift assays (EMSA) and isothermal titration calorimetry (ITC), Eggler et al. (2005) showed that even when challenged with SFN or other chemical activators, the KEAP1:NRF2 complex remains in the 2:1 stoichiometry, with the electrophilic modification of the reactive cysteines of KEAP1 causing no change to the proteins' binding affinity [54]; rather, electrophiles such as SFN cause NRF2 activation by stabilizing the ‘closed’ conformation of the KEAP1:NRF2 complex, effectively trapping KEAP1 to allow newly synthesized NRF2 to enter the nucleus [19]. In the KEAP1-Neh2 complex as predicted by AlphaFold 3 [55], the complex forms even when S40 is phosphorylated (Fig. 3A). Since the S40 phosphate group is predicted to point away from the Kelch domains, no direct steric hindrance effect – or rather, no direct effect on KEAP1 – is predicted to result from S40 phosphorylation.

Early vertebrate evolution observed two rounds of whole-genome duplication; accordingly, many four-member protein families have been identified [56]. This includes the NFE2 family, which comprises of NRF2 alongside NRF1, NRF3, and p45 NF-E2; aside from the similarity in their sequences, their evolutionary association is pronounced due to the proximity of a *HOX* gene to all NFE2 family members [57,58]. The antioxidant response being controlled positively by NRF2/NFE2 and negatively by KEAP1 is a relationship conserved across the vertebrates (with the notable exception of most birds, where NRF2 is active constitutively due to *KEAP1* defects [59]) and in many invertebrate species; the induction of antioxidant defences following treatment with tBHQ was observed in *Drosophila melanogaster*, an insect [60], as well as *Crassostrea gigas*, a mollusc [61]. Yet, when the NRF2/NFE2 sequences are aligned, the ETGE motif is conserved perfectly, and the DLG motif is nearly perfectly conserved (the DMG motif found in echinoderms may present a feasible alternative). Still, the conservation of S40 is incomplete in non-amniote vertebrates and absent from invertebrate sequences (Fig. 3B). From an evolutionary standpoint, the regulation of NRF2 activation by the DLG and ETGE motifs thus far precedes the possible involvement of S40 phosphorylation.

**Fig. 4:**
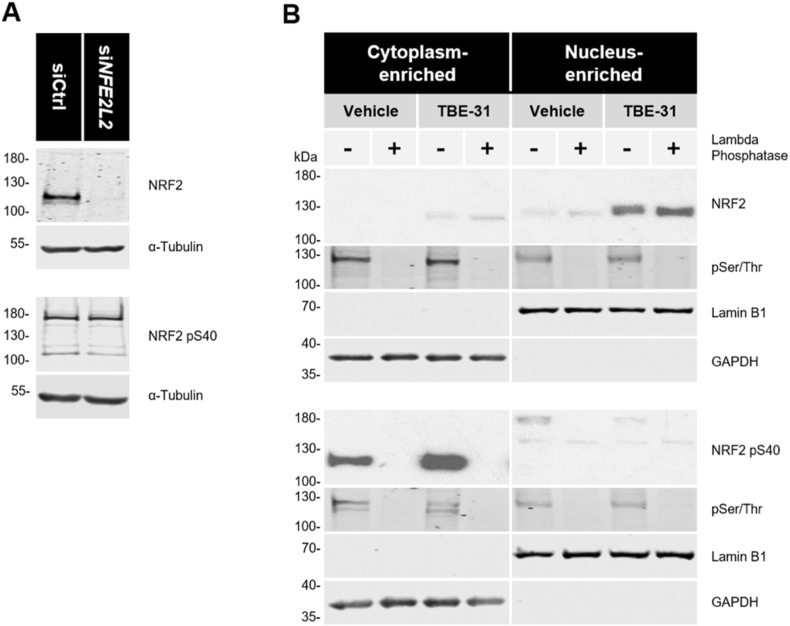
The NRF2 pS40 antibody may not be NRF2 specific. **(A)** ARPE-19 cells were treated with 20 nM siCtrl or si*NFE2L2* for 72 h. **(B)** U2OS cells were treated with 100 nM TBE-31 (an electrophilic NRF2 activator) or 0.1% DMSO (Vehicle) for 1 h, cytoplasm-enriched and nucleus-enriched fractions were separated using 0.5% (v/v) Triton X-100 in 100 mM NaCl, and treated with Lambda Phosphatase for 2 h. The Cell Signaling 20733 and Abcam ab76026 antibodies were used during immunoblotting to detect total and S40-phosphorylated NRF2, respectively.

Lastly, a note of caution regarding the detection of S40 phosphorylation. Point mutants, S40A or S40E [30,35], mimic the constitutive absence or presence of the phosphate group, respectively; however, the expression levels of the ectopically expressed proteins relative to endogenous NRF2 may differ, and glutamate or aspartate may not perfectly represent phosphoserine in all situations [62]. However, most studies investigating S40 phosphorylation, including some cited in this review [33,35], use the Abcam ab76026 antibody to detect pS40. Some studies use the phospho-NRF2 antibody to assess NRF2 activity without accounting for total protein levels, or they interpret phospho-NRF2 as a direct indicator of activation. The authors also tested this antibody. But while some of the immunoblot bands recognized by the antibody migrated similarly to the bands recognized by the antibody against total NRF2, these bands were still present upon NRF2 knockdown, i.e., in the si*NFE2L2* sample, despite the total NRF2 antibody confirming the effectiveness of the knockdown (Fig. 4A) (authors' unpublished observations). Furthermore, following a nuclear-cytoplasmic fractionation, it was observed that the ∼120-kDa ‘NRF2 pS40’ protein was found solely in the cytoplasmic fraction, basally as well as following treatment with the NRF2 activator TBE-31 [63], which caused NRF2 to accumulate in the nucleus (Fig. 4B) (authors' unpublished observations). While Fig. 4B also shows that the epitope recognized by the antibody is indeed a phosphoprotein, and its abundance may be linked to NRF2 activity, the fact that its appearance and behavior differ from those of NRF2 observed with a validated antibody raises doubts about whether the band ascribed to phospho-NRF2 has been correctly identified.

Overall, it is clear that NRF2 is phosphorylated by various kinases. Its S40 may thus be a genuine phosphosite, but the biological context of this modification and its effects on NRF2's affinity for KEAP1 and p21^CIP1^, and ultimately on NRF2's transcriptional activity, remain debatable and require further investigation. The results from many of the experiments addressing the functional significance of NRF2 phosphorylation are ambiguous and therefore difficult to interpret due to the use of unspecific antibodies and non-selective kinase inhibitors. Further experiments employing highly specific reagents and precise molecular manipulations will be essential to prevent imprecise and misleading results and to enable solid conclusions.

### 2.2 Phosphorylation of NRF2 within the Neh6 domain and its modulatory role in β-TrCP interaction

#### 2.2.1 Interaction of NRF2 and β-TrCP/SCF complex

Among the numerous protein partners of NRF2, β-transducin repeat-containing protein (β-TrCP) is constitutively expressed in human cells and is a component of the S-phase kinase-associated protein 1 (SKP1)-Cullin-1 (CUL1)-F-box protein (SCF) complex (Fig. 2C). It plays a crucial role in the ubiquitin–proteosome system for a range of tumor suppressors, oncogenes, and transcription factors [64]. The β-TrCP/SCF complex is a component of a cullin–RING E3 ligase (CRL1), wherein the function of β-TrCP is substrate recognition through its WD40 domain. The WD40 domain is composed of multiple WD40 repeat (WDR) motifs (runs of ∼40 amino acids terminating with a Trp-Asp) that consist of a series of β-sheets and are organized into a 7-bladed β-propeller structure (WDR1-7) that has a shallow substrate binding. It has been demonstrated that the WD40 domain is capable of recognizing consensus degron motifs (DpSGX(1-4)pS) in phosphorylated substrates, leading to their ubiquitination and subsequent proteasomal degradation [65,66]. The final serine residues within the typical degron motifs generally necessitate phosphorylation for effective binding to β-TrCP. The removal of serine residue typically leads to a substantial decrease in binding affinity [65]. This phosphorylation is mediated by GSK3, a serine/threonine kinase that plays an important role in diverse cellular processes, including intermediary metabolism, cell differentiation, proliferation, and development [66].

#### 2.2.2 Mechanism of phosphorylation and binding

The molecular mechanisms of interaction between GSK3 and NRF2 were first described by Rada et al. (2011) [8]. Specifically, GSK3β phosphorylates NRF2 at a serine cluster within its Neh6 domain (DSGIS motif, residues 334-338 in mice), creating a recognition site for β-TrCP, which then targets NRF2 for ubiquitination and proteasomal degradation (Fig. 2C). In KEAP1-null mouse embryonic fibroblasts, the β-TrCP-mediated mechanism remains active. Mutating the DSGIS degron prevented β-TrCP from recognizing NRF2, leading to its increased stability [8]. The follow-up study from the same research group identified two critical serine residues within the DSGIS motif that mediate the interaction with β-TrCP through electrostatic and hydrophobic interactions. Additionally, three key arginine residues in β-TrCP were found to be essential for NRF2 binding. The pharmacological inhibition of GSK3 in mice further demonstrated that preventing β-TrCP-mediated degradation increases NRF2 levels in the liver and hippocampus, leading to an enhanced antioxidant response and reduced oxidative damage [67].

Chowdhry et al. (2013) elucidated a nuanced mechanism by which NRF2 stability is intricately controlled through dual β-TrCP recognition motifs [68]. One of these sites (DSGIS AA 334–338) was regulated by GSK3 phosphorylation, while the other (DSAPGS AA 373–378) was independent of GSK3. These degrons interact with the WDR1-WDR7 repeat domains of β-TrCP to tether the NRF2 protein. Unlike the KEAP1 pathway, where ubiquitination occurs on lysines within the Neh2 domain, β-TrCP1/2-mediated ubiquitination targets lysine residues flanking the Neh6 domain, which lacks intrinsic lysine [68].

The upstream regulation of the GSK3/NRF2/β-TrCP axis is complex and influenced by various cellular conditions and signaling pathways, including the PI3K/AKT pathway. Under basal conditions, GSK3 is constitutively active and is regulated through upstream signaling by inhibition rather than activation. GSK3 exists in two isoforms, GSK3α and GSK3β, both of which are subject to inhibitory phosphorylation by AKT, also known as protein kinase B (PKB), at S21 and S9, respectively [69]. The PI3K/AKT signaling cascade plays a central role in cell survival, growth, and metabolism, primarily activated by growth factors, cytokines, and insulin. Upon ligand binding to receptor tyrosine kinases (RTKs), PI3K is recruited and catalyzes the conversion of phosphatidylinositol-4,5-bisphosphate (PIP2) into phosphatidylinositol-3,4,5-trisphosphate (PIP3), facilitating AKT recruitment. Phosphoinositide-dependent kinase-1 (PDK1) and mechanistic target of rapamycin complex 2 (mTORC2) subsequently phosphorylate AKT at Thr308 and S473, leading to its full activation [69]. Active AKT promotes cell survival by phosphorylating and inactivating pro-apoptotic factors, and while also inhibiting GSK3 activity. Suppression of GSK3 relieves its inhibitory effect on NRF2 (Fig. 2C).

PTEN (Phosphatase and Tensin Homolog) is a lipid phosphatase enzyme primarily known for its activity in the dephosphorylation of PIP3 and reducing its level, thereby suppressing AKT activation. PTEN overexpression results in GSK3 activation [70]. The loss of PTEN showed inhibition of GSK3 and subsequent induction of NRF2 (Fig. 2C), leading to enhanced cholangiocyte expansion in mice [71] and endometrial carcinogenesis in humans [72]. The chemical and genetic targeting of PTEN resulted in GSK3-mediated phosphorylation of NRF2 at residues S335 and S338 and subsequent β-TrCP-dependent but KEAP1-independent degradation [72]. It is evident that loss of PTEN leads to increased NRF2 signature, which provides an advantage for tumor growth [72].

Although the destabilization of NRF2 through β-TrCP partnering is classically considered as a redox-independent regulatory pathway, its upstream signaling components include redox-sensitive proteins, making it susceptible to regulation by electrophilic compounds. Many of these compounds target cysteine residues within the catalytic centers of kinases and phosphatases, altering their activity and indirectly influencing NRF2 stability. For example, electrophilic compounds such as carnosol, tBHQ, triterpenoids, and nordihydroguaiaretic acid have been shown to activate AKT signaling [73]. One key mechanism underlying this activation is the modification of Cys124 in PTEN that negatively regulates the PI3K/AKT pathway and results in sustained signaling [72,73]. Specifically, enhanced AKT activity inhibits GSK3, preventing NRF2 from undergoing β-TrCP-mediated ubiquitination and proteasomal degradation. Collectively, these findings highlight that while the NRF2/β-TrCP axis itself may not be directly redox-sensitive, it remains highly responsive to redox-modulated upstream signals, especially those involving electrophile-sensitive intersections such as PTEN and AKT.

GSK3/NRF2/β-TrCP pathway provides a cooperative degradation mechanism, especially when KEAP1 is dysfunctional, emphasizing its role as an auxiliary system against excessive NRF2 activation. Kuga et al. evidenced this physiological significance of the β-TrCP-mediated degradation pathway by using knock-in mice with a mutant NRF2 that cannot interact with β-TrCP [74]. Under normal conditions, these mutant mice showed no significant differences in NRF2 levels compared to wild-type mice. However, when the KEAP1 level was strongly suppressed, NRF2 accumulated to significantly higher levels in mutant mice, leading to severe hyperplasia and hyperkeratosis in the esophageal epithelium, as well as severe growth retardation. This demonstrates that β-TrCP provides a backup mechanism to prevent uncontrolled NRF2 accumulation when the primary regulator, KEAP1, is compromised [74]. The physiological significance of β-TrCP in NRF2 degradation positions it as a promising target for pharmacological intervention.

#### 2.2.3 Therapeutic targeting of NRF2-GSK3-β-TrCP axis

Based on available research, PHAR appears to be the first and currently only identified small molecule inhibitor capable of selectively disrupting the interaction between β-TrCP and the phosphodegron in NRF2, without affecting KEAP1, through in silico screening of nearly one million compounds [75]. In vitro experiments demonstrated that PHAR induces NRF2 target genes (e.g., *Hmox1*, *Nqo1*, *Gclc*), disrupts the β-TrCP/NRF2 interaction in the cell nucleus, inhibits β-TrCP-mediated ubiquitination of NRF2, and reduces inflammatory cytokine production in macrophages. In the LPS-induced inflammatory model of mice, PHAR selectively targets the liver, greatly attenuating the expression level of inflammatory cytokine genes such as *Il1β*, *Tnf*, and *Il6*. This resulted in a mild and selective activation of NRF2 in liver tissue, approaching normal physiological levels [75]. The subsequent investigation broadened the utilization of PHAR from the domain of inflammation to encompass metabolic dysfunction-associated steatohepatitis (MASH), a prevalent chronic inflammatory liver condition that impedes its functionality and can progress to hepatic fibrosis [76]. Currently, no pharmaceutical agent has received approval specifically for the treatment of MASH. PHAR effectively activated NRF2 in liver cells, including hepatocytes, Kupffer cells, and stellate cells. MRI examination of liver tissue from PHAR-treated STAM mouse model of NASH, based on partial damage to the endocrine pancreas and impairment of insulin secretion, followed by a high-fat diet, revealed that the compound effectively decreased key markers of disease progression, including liver steatosis, hepatocellular ballooning, inflammation, and fibrosis. Transcriptomic analysis revealed that PHAR upregulated anti-fibrotic genes (such as *Plg* and *Bmp7*) while downregulating pro-fibrotic and inflammatory genes, demonstrating a protective effect against NASH progression. Importantly, the compound showed liver-selective activity, suggesting it may be a safer alternative to conventional NRF2 activators [76].

Constitutive activation of NRF2 has been identified as a hallmark of several malignancies, including lung, liver, and pancreatic cancers. In these cases, the sustained activity of NRF2 has been demonstrated to contribute to resistance to therapies and unfavorable clinical outcomes. Consequently, therapeutic strategies that modulate NRF2 stability have emerged as promising anticancer interventions. Although NRF2 “inhibitors” have been extensively investigated, none of the compounds publsihed to date are selective for NRF2. An emerging and innovative alternative involves the use of molecular glue compounds. These are small molecules that enhance specific PPIs rather than disrupting them. This approach offers a key advantage by minimizing off-target effects associated with global NRF2 inhibition. Notably, this strategy preserves the cytoprotective functions of NRF2 in non-tumor tissues. The utilization of “gluing” compounds has been proposed as a strategy to enhance the interaction between KEAP1 and NRF2, as well as between β-TrCP and NRF2, thereby promoting NRF2 ubiquitination and subsequent degradation [77]. This approach aims to counteract the protumorigenic effects of NRF2. Although direct β-TrCP–NRF2 glues have yet to be fully developed, research into molecular glues that stabilize other E3 ligase–substrate complexes (e.g., CRBN and VHL systems), which will be further detailed in Section 3.1.2, has demonstrated feasibility and therapeutic potential [78,79]. Moreover, other reviews have underscored this concept as an underexplored yet promising strategy for modulating NRF2 activity via non-canonical mechanisms [63]. In summary, the stabilization of the KEAP1–NRF2 and the β-TrCP–NRF2 interaction through the use of molecular glues has emerged as a novel therapeutic approach for targeting NRF2-addicted tumors. This approach addresses a significant challenge in the treatment of cancers driven by oxidative stress adaptation and therapy resistance.

### 2.3 Phosphorylations of NRF2 within different Neh domains and their modulatory role in PIN1 interaction

#### 2.3.1 PIN1-NRF2 interaction

There is growing evidence that the peptidyl-prolyl *cis*-trans isomerase NIMA-interacting 1 (PIN1) is an important conditional regulator of NRF2 [80] (Fig. 2D). PIN1 is an 18-kDa protein that catalyzes the *cis*-trans isomerisation of proline residues within numerous substrate proteins. It recognizes phosphorylated serine-proline or threonine-proline (pS/pT-P) motifs within substrates via its N-terminal WW domain, while the C-terminal catalytic, or PPIase, domain is responsible for catalysing prolyl isomerisation [81,82]. Proline isomerisation induces conformational changes to the substrate that modulate protein activity, stability, localization, or its interactions with other biomolecules. PIN1 is involved in the regulation of various physiological processes, including the cell cycle, apoptosis, and differentiation [83]. Notably, it is overexpressed in several cancers and is therefore emerging as a potential therapeutic target [[84], [85], [86], [87], [88]].

PIN1 has been reported as both a positive and negative regulator of NRF2 transcriptional activity. PIN1's role as a positive NRF2 regulator has been associated with a direct phosphorylation-interaction with NRF2, and indirect interactions with other NRF2 regulators that influence its expression levels and cellular localization (Fig. 2D).

#### 2.3.2 Mechanism of phosphorylation and binding

NRF2 is phosphorylated by mitogen-activated protein kinases (MAPKs) at S215, S408, and S577 within the Neh7 domain, the linker region between Neh6 and Neh1, and the Neh3 domain, respectively [10,89,90]. Initially, it was reported that phosphorylation has a minimal effect on NRF2 transcriptional activity and protein levels, leading to only a moderate increase in nuclear localization [89]. However, it later became apparent to have more significant consequences, including direct interaction between NRF2 and PIN1. The first evidence of a direct interaction was obtained via coimmunoprecipitation and immunofluorescence analysis in pancreatic cancer cells [91], where the two proteins were also shown to colocalise by immunofluorescent staining. Similar observations were made in MCF10A-Ras mammary epithelial cells, where the interaction was also verified using a proximity ligation assay. However, these studies did not associate the interaction with NRF2 phosphorylation. This link was later elucidated through co-immunoprecipitation and proximity ligation experiments in MDA-MB-231 breast cancer cells. Treating the cells with JNK and ERK kinase inhibitors (but not p38 inhibitors) substantially diminished the interaction, as did the introduction of alkaline phosphatase to HEK-293 cells. Thus, the phosphorylation dependence of the NRF2-PIN1 interaction was confirmed.

All three phosphosites are crucial for the interaction, which is barely detectable in HEK-293 cells expressing NRF2 with single-point mutations at S215A, S408A, and S577A, respectively [90]. This is intriguing because in vitro fluorescence polarization experiments conducted by Ozleyen et al. (2025) have shown that short phospho-peptide mimics of each of the motifs bind to PIN1 with physiologically relevant apparent dissociation constants (*K*_d_) of 157, 221 and 168 nM, respectively [92]. Indeed, the NRF2 motifs bind to PIN1 with higher affinity than well-characterised substrates such as Tau, which bind with *K*_d_s in the micromolar range [93]. One explanation for this could be that the short phospho-peptide mimics do not take into account the 3-dimensional conformation and/or conformational dynamics of full length NRF2. In this context, access to the phosphosites for PIN1 binding might be restricted and, therefore, PIN1 needs to sample multiple binding sites. Alternatively, NRF2 might engage with PIN1 in a multivalent fashion [94]. Mutation of PIN1 in the WW domain (W34A) or PPIase domain (K63A) abolished its interaction with NRF2 [90]. Computational simulations have predicted that the three phospho-peptide mimics can interact with the PIN1 WW-domain [92]. However, further studies are needed at the molecular level to elucidate the binding mechanism fully, determine if PIN1 catalyzes proline isomerisation, and investigate the structural consequences of this process.

Interestingly, recent findings by Saeidi et al. (2022) indicate that PIN1 may also interact with phosphorylated residues on KEAP1, specifically S104 and T277 [90]. While the precise functional consequence of this binding remains to be fully elucidated, it is postulated that PIN1 may compete with NRF2 for KEAP1 binding, effectively sequestering free KEAP1 and reducing its capacity to mediate NRF2 degradation. Such a mechanism would amplify NRF2 signaling through a dual effect: stabilizing NRF2 directly and impairing KEAP1's repressive function. However, beyond its canonical role in NRF2 repression, KEAP1 possesses a broad interactome. It is functionally implicated in diverse cellular processes, including the regulation of proteostasis, cell cycle progression, cytoskeletal organization, nitric oxide biology, and S-nitrosation pathways [95]. Therefore, further studies are needed to clarify the molecular details and functional significance of the PIN1–KEAP1 interaction.

Moreover, the known PIN1-binding sites on NRF2—S215, S408, and S577—are located within or adjacent to the Neh7, Neh6, and Neh3 domains, which are critical for interactions with other context-dependent regulators such as RXRα/RARα, β-TrCP, and chromodomain helicase DNA-binding protein 6 (CHD6). It is therefore plausible that PIN1 binding may sterically hinder or compete with these factors, shifting the regulatory landscape in favor of NRF2 stabilization and activation. Further investigation is warranted to explore whether PIN1 selectively modulates NRF2's interactome at these sites to promote its oncogenic function.

#### 2.3.3 PIN1 as a positive and negative regulator of NRF2

In cancer cells, PIN1 increases NRF2 transcriptional activity by stabilizing NRF2 protein levels and facilitating nuclear translocation. For example, in cervical cancer cells, PIN1 enhances NRF2 expression at both the mRNA and protein levels. This results in increased NRF2-mediated expression of glutathione peroxidase 4 (GPX4), which protects cells from oxidative stress and promotes the survival of cancer cells [96].

In pancreatic cancer cells, PCR arrays, luciferase reporter assays, and qPCR showed that PIN1 upregulated the expression of NRF2 target genes. This results from PIN1 and NRF2 occupying the same location on the ARE region of the *HMOX1* promoter, as elucidated in a re-ChIP assay. Furthermore, Western blot and immunofluorescence experiments showed that PIN1 promoted the nuclear accumulation of NRF2 [91]. The physiology underpinning PIN1 activity is highly complex – even the physical properties of pancreatic adenocarcinoma cells influence PIN1 expression and the synergistic activation of NRF2 transcriptional activity [97]. Ultimately, high levels of PIN1 and NRF2 were shown to be predictive of unfavorable prognosis in patients with the disease [91].

In breast cancer cells (MCF-7) and human mammary cells (MCF10A-Ras), PIN1 does not affect NRF2 mRNA levels but does increase NRF2 protein levels [90,98]. The enhancement of PIN1-induced NRF2 expression is abrogated by treatment with the PIN1 inhibitor all-trans retinoic acid (ATRA). Silencing PIN1 in MDA-MB-231 cells using siRNA results in elevated rates of NRF2 degradation, likely due to an increase in its ubiquitination [90]. Ubiquitination was further induced upon treatment with ATRA, but the resulting degradation was reversed by treatment with the proteome inhibitor MG-132 [98]. PIN1 was also shown to promote NRF2 nuclear translocation accumulation in MCF10A-Ras [98] and MDA-MB-231 cells, an effect that is again abrogated upon treatment with ATRA [90]. It has been proposed that PIN1-mediated NRF2 nuclear translocation might involve importin-α5 and the dynein motor complex [99]. In terms of breast cancer tumor growth, PIN1 siRNA reduces colony formation, attenuates invasiveness, and decreases the migratory capability of MDA-MB-231 cells. Furthermore, mice inoculated with MDA-MB-231 cells treated with PIN1 siRNA had reduced levels of NRF2 and developed smaller tumors [90].

The direct interaction of PIN1 with KEAP1 also stabilizes NRF2 in MDA-MB-231 cells. The interaction is dependent on the phosphorylation of KEAP1 at S105 and T277, and hampers KEAP1 E3 ligase activity. It was postulated that PIN1 and KEAP1 compete for binding to NRF2 because the PIN1/NRF2 complex was more abundant in KEAP1 knock-out MEFs [90]. This suggests that PIN1 is involved in a broader, complex system of regulatory checks that control NRF2 activity.

In different physiological and pathological conditions, PIN1 has been characterised as a negative regulator of NRF2. In particular, this is the case in the regulation of NRF2/HMOX1. For example, PIN1 has been shown to exacerbate renal ischemia-reperfusion injury in rats by downregulating the NRF2/HMOX1 pathway [100]. A similar observation was made in vascular smooth muscle cells, where PIN1 also diminished NRF2 nuclear localization [101]. In mouse embryonic fibroblasts (MEFs), PIN1 reduced NRF2 transcriptional activity, and in these NIH3T3 cells, PIN1 overexpression resulted in increased NRF2 ubiquitination and decreased protein levels [101]. In both these cases, the negative regulation of NRF2 by PIN1 was not directly linked to NRF2 phosphorylation or an NRF2-PIN1 interaction. Thus, PIN1 is likely exerting these regulatory effects via interactions with other proteins. The indirect regulation of NRF2 transcriptional activity by PIN1 was highlighted in another study where PIN1 reduced the expression of NRF2 target genes (*NQO1*, *GSTA1*) in MEFs, in response to oxidative stress [102]. This effect was partially attributed to PIN1 directly interacting with and inhibiting the phosphatidylinositol-5-phosphate 4-kinase (PIP4α) in a p38-induced phosphorylation-dependent manner [102]. This reduced the abundance of phosphatidylinositol-5-phosphate (PtdIns5P), which is required for the cellular response to oxidative stress and promotes NRF2-mediated transcription.

#### 2.3.4 Therapeutic targeting of PIN1-NRF2 interactions

Compared to the extensive studies investigating NRF2 interactions with its canonical repressors KEAP1 and β-TrCP, pharmacological efforts targeting the PIN1–NRF2 interaction remain relatively limited. Recent biophysical work by Ozleyen et al. (2025) has shed light on the effects of small molecule PIN1 inhibitors—juglone, KPT-6566, and epigallocatechin gallate (EGCG)—on this protein–protein interaction [92]. Fluorescence polarization assays revealed that juglone, a covalent PIN1 inhibitor, moderately decreased the binding affinity between PIN1 and phosphorylated NRF2 peptides (S215, S408, S577), with IC_50_ values ranging from 20 to 30 μM [92]. In contrast, KPT-6566 exhibited markedly greater potency, reducing binding affinity by up to 149-fold and demonstrating submicromolar IC_50_ values (0.3–1.4 μM). Mechanistic studies suggest that KPT-6566 acts via conjugate addition, forming a covalent bulky KPT-6566-B adduct that disrupts the PIN1–NRF2 interaction, likely through allosteric modulation of the WW domain [92]. Surprisingly, EGCG—despite its previously reported PIN1-inhibitory effects—showed no measurable disruption of the PIN1–NRF2 interaction under the same conditions. These findings highlight the therapeutic promise of KPT-6566 as a potent and mechanistically distinct inhibitor of PIN1-mediated NRF2 stabilization, although further validation in cellular and in vivo models remains necessary [92].

In addition to these small molecules, the clinically approved compound all-trans retinoic acid (ATRA) has also been reported to inhibit the activity of both NRF2 [103] and PIN1 [90]. In breast cancer cells, ATRA effectively abrogated the nuclear accumulation of NRF2 and blocked its stabilization by PIN1 [90]. While these results suggest a potential role for ATRA in suppressing NRF2 function through PIN1 inhibition, it is important to note that no direct biophysical evidence currently supports this mechanism. Specifically, whether ATRA disrupts the PIN1–NRF2 PPI remains unresolved. This highlights a key gap in the literature and underscores the need for future mechanistic studies utilizing direct biophysical approaches to validate the PIN1-dependence of ATRA's effects on NRF2 [90]. In any case, pharmacological inhibition of PIN1 with small molecules such as ATRA, juglone and KPT-656 shows great promise as a strategy for modulating NRF2 activity in disease [90]. Further structural and functional studies are now needed to inform the rational design of more selective and potent inhibitors.

## 3 NRF2-partner protein interaction modulating its post-translational modifications

The function of NRF2 is tightly regulated by a network of partner proteins that modulate its PTMs, consequently affecting its stability and transcriptional efficiency. Different partners can either promote NRF2 degradation through ubiquitination or boost its transcriptional activity through activating modifications, such as acetylation. This section highlights key NRF2-binding partners: KEAP1, WDR23, and CBP exemplify how PPIs control the direction of NRF2's PTMs.

### 3.1 The KEAP1-NRF2 interaction facilitates the ubiquitination of NRF2

#### 3.1.1 Mechanism of PPI and ubiquitination

As mentioned above in detail (section 2.1.1) in the canonical KEAP1-dependent degradation pathway, NRF2 initially engages KEAP1 via the high-affinity ETGE and low-affinity DLG motifs, thereby enabling CUL3-mediated ubiquitination and continuous proteasomal turnover [104]. All BTB-Kelch family proteins, including KEAP1, function as substrate recognition units and binding subunits for Cullin-RING ligases (CRLs). The BTB domain mediates both KEAP1 homodimerization and its interaction with the N-terminal domain of CUL3. Each monomer binds one molecule of CUL3, forming a symmetric complex. This interaction is further stabilized by a conserved 3-box motif within the IVR region, which forms a hydrophobic groove accommodating the N-terminal extension of CUL3 [104,105].

**Fig. 5:**
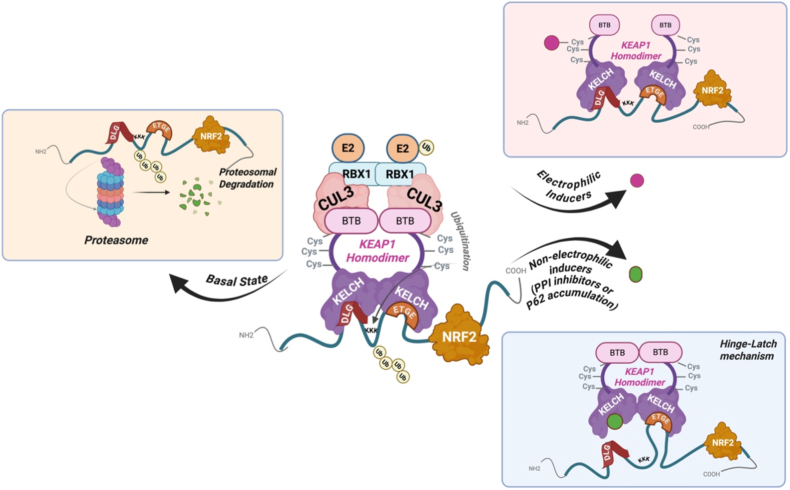
Structural modelling of KEAP1-NRF2. In the canonical model, NRF2 binds the KEAP1 homodimer through its high-affinity ETGE “hinge” and low-affinity DLG “latch” motifs, positioning lysine residues for CUL3-RBX1–mediated ubiquitination and proteasomal degradation. The CUL3–RBX1 E3 ligase complex, stabilized by KEAP1's BTB and 3-box domains, facilitates Lys48-linked polyubiquitin chain formation on NRF2. Oxidative or electrophilic modification of KEAP1 cysteines (e.g., Cys151, Cys273, Cys288) disrupts this ubiquitination machinery, allowing NRF2 stabilization and nuclear translocation. Non-electrophilic activation mechanisms including p62 accumulation and small-molecule PPI inhibitors primarily target the hinge-and-latch configuration to prevent KEAP1-dependent ubiquitination.

CUL3 serves as a scaffold within the CRL complex, linking the substrate adaptor KEAP1 at its N-terminal domain with the RING-box protein RBX1 at its C-terminal end. RBX1 (also known as ROC1) provides a docking site for the E2 ubiquitin-conjugating enzyme, which is loaded with activated ubiquitin (Fig. 5) [106,107]. The activity of the CUL3-RBX1 complex is regulated by neddylation, a post-translational modification involving the covalent attachment of ubiquitin-like protein NEDD8 to a conserved lysine residue in the CUL3 C-terminal domain. Neddylation induces a conformational change that liberates the RING domain of RBX1 from its inhibitory interaction with the Cullin scaffold, enhancing its flexibility and catalytic efficiency [108].

Mechanistically, KEAP1/NRF2 complex utilizes “hinge and latch model” as one of the mechanism where high affinity ETGE motif acts as a stable hinge, forming strong electrostatic and hydrogen bond interactions between NRF2 and KEAP1 (Fig. 5). On the other hand, the low-affinity DLG motif serves as a more dynamic latch, stabilizing the orientation of NRF2 between two monomers. This arrangement exposes seven lysine residues that connect the ETGE and DLG motifs, facilitating efficient ubiquitination [109,110]. Ubiquitination is a multi-step process involving the sequential action of E1 ubiquitin-activating enzyme, E2 ubiquitin-conjugating enzyme and E3 ubiquitin ligase. First, ubiquitin is activated by E1 through the formation of a high-energy thioester bond with its catalytic cysteine residue. The activated ubiquitin is then transferred to the catalytic cysteine of an E2 ubiquitin-conjugating enzyme. The E3 ligase, specifically the KEAP1–CUL3–RBX1 complex, binds both the E2-ubiquitin and the substrate NRF2, facilitating the transfer of ubiquitin from the E2 to lysine residues on NRF2, forming an isopeptide bond [111].

This initial monoubiquitination is followed by the successive addition of ubiquitin molecules to form a K48-linked polyubiquitin chain. Once the polyubiquitin chain reaches at least four ubiquitins, NRF2 is recognized as a substrate by the 26S proteasome and targeted for degradation (Fig. 5). This ensures low basal levels of NRF2 under homeostatic conditions, allowing for tight control over the expression of cytoprotective genes [112].

Upon exposure to oxidative stress or electrophilic compounds, specific cysteine residues on KEAP1 - such as C151, C273, and C288 - undergo modifications (oxidation, alkylation, or S-nitrosation). These modifications disrupt the structural integrity of KEAP1, resulting in a conformational change that impairs its ability to tether NRF2 and form a functional ubiquitin ligase complex. Consequently, newly synthesized NRF2 escapes ubiquitination, accumulates in the cytoplasm, and translocates into the nucleus. There, NRF2 heterodimerizes with Maf proteins via its Neh1 domain and activates the transcription of antioxidant response element (ARE)-driven genes involved in detoxification, redox balance, and metabolic homeostasis [113]. In contrast to this cysteine-based sensing mechanism, the hinge-and-latch configuration of KEAP1–NRF2 is preferentially exploited by non-electrophilic modes of NRF2 activation, such as the autophagy adaptor p62 and pharmacological KEAP1–NRF2 protein–protein interaction inhibitors, whereas classical electrophilic NRF2 inducers do not primarily act through this hinge-and-latch mechanism (Fig. 5) [114].

#### 3.1.2 Therapeutic targeting of NRF2-KEAP1-CUL3 axis

The NRF2-KEAP1-CUL3 axis has emerged as an attractive therapeutic target in diseases associated with oxidative stress and inflammation. Pharmacological inhibition of KEAP1, either by covalent modification of its reactive cysteines or by competitive binding to its Kelch domains, leads to NRF2 stabilization and enhanced activity. Alternative strategies include disrupting interactions between KEAP1 and CUL3, targeting BTB domain of KEAP1, the C-terminal domain of CUL3 or KEAP1 3-box motif may also destabilize this interaction. Moreover, targeting E1-E2-E3 enzymes involved in the ubiquitination cascade and its interactions represents another possible route for controlling NRF2 activity [115]. Hundreds of natural products and synthetic molecules have been identified or designed to modulate NRF2-KEAP1 pathway. Electrophilic KEAP1 inhibitors remain the dominant category, though non-covalent inhibitors are also advancing [3]. To date, only two KEAP1-targeting drugs have been approved for clinical practice: dimethyl fumarate (DMF) [116,117] and omaveloxolone [[118], [119], [120]]. Both compounds are electrophiles that covalently modify sensor cysteine residues within KEAP1, primarily C151 in the BTB domain [3].

##### 3.1.2.1 Electrophilic KEAP1 inhibitors

DMF induces expression of NRF2-target genes, such as NQO1 in human peripheral blood mononuclear cells [121]. It demonstrated efficiency in placebo-controlled phase III clinical trial for relapsing-remitting multiple sclerosis (RRMS), leading to FDA approval in 2013 (Tecfidera, Biogen) [116,117]. It subsequently gained approval (Skilarence®) in Germany in 2017 for the systemic treatment of moderate to severe plaque psoriasis [122]. Additional fumarate derivatives have followed, like diroximel fumarate (DRF; Vumerity®, Biogen) or monomethyl fumarate (MMF; Bafiertam®, Banner Life Sciences) and in recent years they have also been approved for clinical use for multiple sclerosis with improved gastrointestinal tolerability [123]. Fumarates continue to be evaluated in diverse indications including ALS, rheumatoid arthritis, pulmonary hypertension, intracranial aneurysm and several cancers [122].

Omaveloxolone, a synthetic oleanane triterpenoid, became the first approved drug for Friedriech's ataxia in 2023 (Skyclarys®) [119,120,124]. Bardoxolone methyl (CDDO-Me), the parent molecule, reached phase II clinical trials for chronic kidney disease, but the study was terminated due to cardiovascular safety concerns [125]. Nonetheless, both CDDO-Me and omaveloxolone remain under investigation in clinical trials for diseases including breast cancer, mitochondrial myopathy, pulmonary hypertension, type II diabetes and liver disease [3].

Other, naturally occurring, electrophilic NRF2 inducers are also under investigation. The best characterized among them, SFN, an isothiocyanate isolated from broccoli through its isothiocyanate (-N=C=S) group easily binds with KEAP1 sensor cysteines [126]. Mainly C151 in the BTB domain, but in some cases also with C38, C368 and C489. The compound has demonstrated efficacy across numerous pre-clinical models pertaining to chronic diseases [[127], [128], [129]].

Electrophiles do not directly disrupt KEAP1-NRF2 binding, instead, modification of KEAP1 sensor cysteines alters KEAP1 conformation and CUL3 recruitment leads to accumulation of an inactive KEAP1-NRF2 complex. In this “hinge-latch” disabled state, KEAP1 is not regenerated, allowing newly synthesized NRF2 to escape degradation [19,110]. Certain electrophiles, such as N-iodoacetyl-N-biotinylhexylenediamine (IAB), have been shown to impair KEAP1-CUL3 binding and reduce NRF2 ubiquitination through C151 modification [130].

##### 3.1.2.2 Non-covalent KEAP1 inhibitors

Non-covalent KEAP1 inhibitors are designed to occupy Kelch domain that recognizes ETGE and DLG motifs of NRF2. By competitively displacing NRF2 from these binding motifs, these molecules block NRF2 ubiquitination, leading to its cytoplasmic stabilization, nuclear translocation and transcriptional activation [110]. Non-covalent KEAP1 inhibitors are conceptually more attractive due to their target selectivity, yet they face major chemistry challenges. They must bind a deep, highly polar arginine-rich pocket of KEAP1 essential for NRF2 recognition, while maintaining membrane permeability and metabolic stability. Current research focuses on studying several compounds, mostly derivatives of tetrahydroisoquinoline (THIQ), phenylpropanoic acid derivatives or macrocycle compounds. Despite substantial structural progress, no non-covalent KEAP1 inhibitor has yet advanced into clinical trials [3].

##### 3.1.2.3 Cullin 3 inhibition

Neddylation of CUL3 is essential for full activation of the CUL3–RBX1 E3 ligase complex. This PTM is catalyzed by the NEDD8-activating enzyme E1 (NAE1) and facilitated by DCN1 scaffold proteins [3]. Pharmacological inhibition of this pathway via NAE1 inhibitors (e.g., MLN4924/pevonedistat) or DCN1 blockers, reduces E3 ligase activity, impairs KEAP1-mediated NRF2 ubiquitination, and robustly activates NRF2 signaling [[131], [132], [133]].

##### 3.1.2.4 NRF2 inhibitors

Constitutive activation of NRF2 is a hallmark of several cancers, often driven by loss-of-function KEAP1 mutations or gain-of-function mutations in the ETGE/DLG motifs of NRF2. Additional epigenetic and post-transcriptional changes contribute to sustained NRF2 signaling [134]. Hyperactive NRF2 enhances cancer cell proliferation, rewires metabolism, suppresses oxidative stress, modifies cell death pathways, and promotes immune evasion [135,136]. Blocking NRF2 activity in such tumors represents a therapeutic opportunity.

Early NRF2-lowering compounds, such as brusatol, febrifugine, and halofuginone show non-specific inhibition and act beyond the KEAP1 axis, limiting their clinical relevance [137,138]. MSU38225 induces NRF2 degradation independently of both KEAP1 and β-TrCP, highlighting an alternative strategy for NRF2 suppression [139,140]. **Pro**teolysis **Ta**rgeting **C**himera (PROTAC) technology has introduced new possibilities. PROTACs consist of a ligand for the target protein, a ligand for an E3 ligase, and a linker bringing both into proximity to induce ubiquitination and proteasomal degradation. KEAP1 has been used as an E3 ligase component to degrade other proteins [141,142]. More recently, ARE-derived PROTACs (ARE-PROTACs) were designed to degrade NRF2 by recruiting the NRF2–MAFG complex via ARE sequences, sensitizing NRF2-hyperactive NSCLC cells to ferroptosis [143]. VVD-065 exemplifies a different strategy. Instead of inhibiting KEAP1, VVD-065 covalently modifies Cys151 to increase KEAP1–CUL3 affinity, enhancing NRF2 ubiquitination and promoting NRF2 degradation [77]. This mode of action makes VVD-065 one of the few direct NRF2 inhibitors with clinical promise. Its derivative, VVD-130037, has recently entered phase I clinical trials for advanced solid tumors (clinicalTrials.gov ID: NCT05954312) [144].

### 3.2 The WDR23-NRF2 interaction promotes the ubiquitination of NRF2

**Fig. 6:**
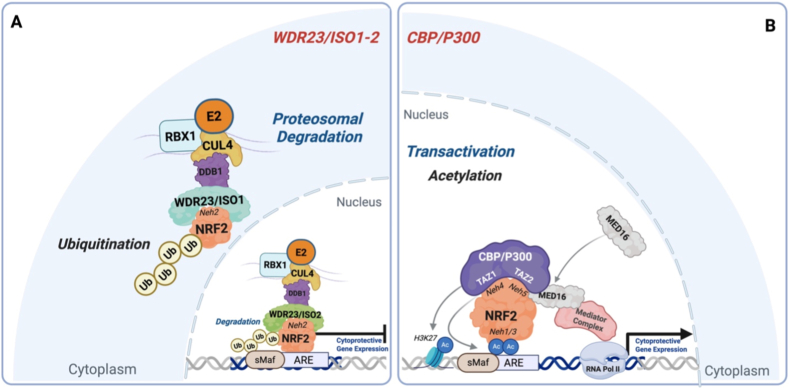
Modulation of NRF2 PTMs by WDR23 and CBP/p300 partners. **(A)** WDR23 isoforms regulate NRF2 stability through distinct subcellular mechanisms: WDR23/ISO1 in the cytoplasm and WDR23/ISO2 in the nucleus recruit the DDB1–CUL4 ubiquitin ligase complex, promoting NRF2 ubiquitination and degradation. **(B)** CBP/p300 interacts with the Neh4/5 transactivation domains of NRF2 via their TAZ1/TAZ2 domains, enhancing NRF2 transcriptional activity through acetylation of residues within the Neh1/2 region.

To the best of our knowledge, under basal conditions, the NRF2 protein is maintained at low cellular levels through KEAP1/CUL3-mediated proteasomal degradation. Although KEAP1 exerts its regulatory function mainly in the cytoplasm, it has long been proposed that NRF2 may also be subject to additional layers of regulation within the nucleus [[145], [146], [147]] (Fig. 6). WDR23, also known as DDB1- and CUL4-associated factor 11 (DCAF11), was identified as a functionally and evolutionarily conserved regulator of NRF2 that promotes its degradation in a comparative study using *C. elegans* and human cell culture models [148]. In mammals, two WDR23 isoforms are expressed in both cytoplasmic and nuclear fractions. Isoform 1 (UniProtKB/Swiss-Prot Accession: Q8TEB1-2) is primarily localized in the cytoplasm, whereas isoform 2 (UniProtKB/Swiss-Prot Accession: Q8TEB1-1) is enriched in the nucleus but can also be detected in the cytoplasm upon overexpression. These distinct subcellular distributions suggest that WDR23 may regulate NRF2 at both cytoplasmic and nuclear levels [148] (Fig. 6A).

Although studies on WDR23-mediated regulation of NRF2 are limited, Lo et al. (2017) first identified WDR23 as an alternative regulator of NRF2 proteostasis [148]. WDR23 functions as a substrate receptor for the CUL4–DDB1 E3 ubiquitin ligase complex, suppressing NRF2 activity in a KEAP1-independent manner. Structurally, WDR23 contains seven WD40 repeats that facilitate substrate recognition and protein–protein interactions, enabling its direct association with the CUL4–DDB1 complex and its role in ubiquitination and proteasomal degradation [149].

Immunoprecipitation assays using GFP-tagged WDR23 isoforms and HA-tagged NRF2 confirmed that both isoforms co-immunoprecipitate with NRF2, indicating complex formation. WDR23 also efficiently interacted with DDB1 and CUL4A, but not KEAP1, and its silencing restored NRF2 levels in HEK-293T cells [148]. Treatment with the proteasome inhibitor MG-132 prevented WDR23-mediated NRF2 reduction, confirming that NRF2 degradation occurs through the ubiquitin–proteasome pathway. In vitro, the purified WDR23–DDB1–CUL4 complex efficiently ubiquitinated NRF2, which also supports this mechanism [148].

Overexpression of WDR23 markedly reduced the nuclear accumulation of newly synthesized NRF2 in H_2_O_2_-treated cells, without altering KEAP1 expression levels [148]. These findings indicate that WDR23 regulates NRF2 stability through a KEAP1–CUL3-independent pathway. Further domain-mapping analyses using a panel of NRF2 deletion mutants demonstrated that loss of the Neh2 domain (ΔNeh2) completely abolished WDR23 binding. In contrast, deletion of other Neh domains did not affect the interaction [148]. This observation identifies the Neh2 domain as essential for WDR23–NRF2 association. Since KEAP1 also targets NRF2 through the Neh2 domain, these results suggest that both proteins recognize overlapping structural regions but likely engage distinct binding motifs. To further define this interaction, truncated NRF2 mutants Neh2A (Δ2–43) and Neh2B (Δ44–86) were examined. WDR23 bound to Neh2B but not to Neh2A, indicating that the N-terminal portion of the Neh2 domain mediates WDR23 recognition. Notably, this region contains the DIDLID sequence, distinct from the DLG and ETGE motifs that are essential for KEAP1 binding. Consistent with this, mutation of either DLG or ETGE did not disrupt WDR23–NRF2 interaction, confirming that WDR23 binds NRF2 independently of KEAP1. These findings collectively suggest that WDR23 acts as an alternative adaptor within the ubiquitin–proteasome system, targeting NRF2 through a unique Neh2-binding interface and maintaining its degradation under conditions where KEAP1-mediated regulation may be impaired [148].

To assess the functional consequences of this regulation, Lo et al. (2017) treated WDR23-overexpressing HEK-293T human embryonic kidney cells with chemotherapeutic agents, including etoposide, doxorubicin, and cisplatin. WDR23 overexpression increased cellular sensitivity to chemotherapy, as evidenced by enhanced DNA damage and apoptosis, highlighting WDR23 as a potential therapeutic target in cancer [148].

More recently, WDR23 has also been implicated in metabolic regulation. Genetic deletion of Wdr23 in mice resulted in increased expression and activity of the insulin-degrading enzyme (IDE), leading to reduced plasma insulin levels and impaired insulin sensitivity in males [150]. Similar findings were observed in WDR23(−/−) human hepatocytes, which exhibited phosphorylation defects in insulin signaling pathways, including AKT, MAPK, FOXO1, and mTOR. Loss of WDR23 stabilized NRF2, leading to elevated IDE expression and metabolic imbalance due to excessive insulin degradation. Silencing NRF2 restored IDE levels, confirming the regulatory link between WDR23 and NRF2 [150]. Collectively, these findings suggested that the WDR23–NRF2 axis may serve as an important biomarker and therapeutic target in both cancer and metabolic diseases.

### 3.3 The CBP-NRF2 interaction drives NRF2 acetylation

CREB-binding protein (CBP) and p300 are closely related transcriptional coactivators possessing intrinsic histone acetyltransferase (HAT) activity and share a high degree of structural homology. By acetylating histones, they relax chromatin structure and facilitate transcription by recruiting the basal RNA polymerase machinery [151,152]. Beyond histones, numerous non-histone proteins, particularly transcription factors, serve as substrates for CBP/p300, thereby expanding their regulatory influence on gene expression and modulating substrate stability, localization, and activity. Among these substrates, the transcription factor NRF2 has emerged as a key target of CBP/p300-mediated acetylation (Fig. 6B).

CBP/p300 interacts directly with NRF2, enhancing its binding to ARE promoters through acetylation and consequently promoting the transcription of downstream cytoprotective genes [[153], [154], [155], [156]]. Early studies demonstrated that acetylation of the bZIP domain of MafG by CBP facilitates the DNA-binding ability of the NRF2–MafG heterodimer [153]. Given that MafG is also a core binding partner of NRF2, it was proposed that recruitment of CBP/p300 to AREs by NRF2 could enable the acetylation of small Maf proteins as well. These observations highlight a cooperative model in which CBP/p300-mediated acetylation of both NRF2 and MafG synergistically enhances DNA binding and transcriptional activation. NRF2, which stabilizes under oxidative stress conditions, translocates to the nucleus, forms heterodimers with sMAF proteins, and binds to ARE sequences [157]. NRF2 directs CBP/p300 to the Neh4 and Neh5 transactivation regions, thereby facilitating the acetylation of lysine residues in its own Neh1 and Neh3 regions as well as H3K27 in the surrounding chromatin. Through Neh 4 and 5, it interacts with MED16 to recruit the mediator complex and RNA Pol II to transcription start sites [158]. This coordinated mechanism induces the expression of antioxidant genes such as *SLC7A11*, *GCLC*, *GCLM*, *GPX2*, and *TXNRD1*, thereby reducing ROS levels and protecting the cell from apoptosis and ferroptosis [159] (Fig. 6B).

More recently, Ganner et al. (2020) confirmed the physical interaction between p300 and NRF2 and demonstrated that p300-mediated acetylation increases NRF2 protein stability [156]. The D1399Y mutation, which abolishes the acetyltransferase activity of p300, prevented NRF2 stabilization, underscoring the requirement for HAT activity in this effect. Mechanistically, p300 disrupts the KEAP1–NRF2 interaction, thereby protecting NRF2 from KEAP1-mediated proteasomal degradation. This stabilization occurs post-translationally, as silencing of p300 reduced NRF2 protein levels without affecting mRNA expression. Functionally, p300 overexpression enhanced cellular survival under H_2_O_2_-induced oxidative stress, whereas shRNA-mediated knockdown impaired HO-1 induction, confirming the role of p300 in NRF2-dependent antioxidant defense [156].

The molecular interaction between NRF2 and CBP/p300 was first elucidated through a yeast two-hybrid screening. This study demonstrated that binding of the CBP TAZ1 and TAZ2 domains to the NRF2 Neh4 and Neh5 domains is essential for NRF2 to achieve maximal transactivation [160]. Sun et al. (2009) later identified that multiple lysine (K) residues in the Neh1 DNA-binding domain of NRF2 are key sites for acetylation [155]. Notably, replacing these residues with arginine (K3R) did not impact NRF2 protein stability, a result explained by the Theorell–Chance catalysis model. According to this model, p300 catalyzes acetylation without forming a tight complex with its substrates; lysine residues pass through the catalytic tunnel of p300, where they react with acetyl-CoA. This mechanism accounts for the remarkable flexibility of the enzyme and its ability to acetylate a broad spectrum of substrates possessing multiple acetylation sites, including p53, c-Myc, FoxOs, and STAT proteins [155]. Other studies have shown that lysine residues in specific regions of the NRF2 protein contribute to its transcriptional activation potential through acetylation. Kawai et al. (2011) demonstrated that mutation of K588 and K591 residues in the Neh3 region to alanine or arginine markedly reduced NRF2-dependent *HMOX1* reporter activity [154]. Moreover, these mutations completely revoked the ability of CBP to enhance the transcriptional activity observed with wild-type NRF2. These findings indicate that these lysine residues are critical acetylation sites required for efficient CBP interaction. Furthermore, it has been emphasized that acetylation occurring at different structural domains of NRF2 cooperatively regulates its transcriptional activity. Collectively, these results suggest that, in addition to the previously characterized Neh4 and Neh5 domains [160], the Neh3 domain also makes a significant contribution to the transactivation capacity of NRF2.

Recent studies have further underscored the importance of the Neh4 and Neh5 regions in NRF2 transcriptional activity [161]. These domains enhance transcriptional activation by directly interacting with the TAZ1 and TAZ2 regions of CBP/p300. Biophysical analyses revealed that Neh4 and Neh5 are intrinsically disordered regions that undergo conformational transitions upon binding to CBP/p300. NMR studies have shown that, while these regions exhibit high flexibility in isolation, they adopt defined secondary structures during complex formation [161]. Notably, CBP/p300 associates with numerous transcription factors, and in several instances, competitive binding occurs between NRF2 and other factors such as HIF-1α, CITED2, and STAT1. This competition for shared CBP/p300 binding sites is thought to adjust NRF2 transcriptional activity in response to different cellular signals [161].

## 4 Unresolved mechanisms of NRF2-Partner protein interactions and post-translational modifications

Despite the extensive work to characterize either the canonical or noncanonical PPI axis and the several established PTM-mediated regulatory routes, as detailed above, many NRF2-partner protein interactions and their associated PTMs remain incompletely understood (Table 1, Table 2). Most studies in this area date back to the early 2000s, and there is a notable lack of biophysical studies that detail site-specific interactions or provide mechanistic insights. Besides, it remains unclear whether these PPIs require specific PTMs to occur or if these interactions themselves trigger subsequent PTMs. Therefore, the following subsections address these less-defined partners and atypical PTMs by presenting key gaps and open questions in the field.

**Table 1:** NRF2 conditional partners.

Interacting Protein	Interacting Sites on Partner Protein	Interacting Sites of NRF2 (Domains)	Used Models	Ultimate Effect	References
MED16	N-terminal region of MED16	Neh4/5 Neh1 (with less capability)	*PTEN/KESP1 mutant mouse models for endogenous PPIs detection Hepa1c1c7 mouse hepatoma cells* for creating Med16 knockout cells performing microarray/gene expression analysis *293F human embryonic kidney cells* for biochemical purification of the NRF2-Mediator complex *Hep3B human hepatoma cells* for MED16 siRNA knockdown *A549 human lung adenocarcinoma cells* for ChIP assays	Direct association with NRF2 serves as a conduit to transmit NRF2-activating signals to RNA polymerase II. Deficiency of MED16 reduced the expression of ∼75% of NRF2-dependent antioxidant genes expression	[158]
BRG1	Actin-binding site of BRG1 (assumption)	Neh5	*SW480 human colon carcinoma cells* for NRF2/BRG1 siRNA and shRNA silencing and ChIP Assays SW13 human adrenal carcinoma cells for BRG1 reconstitution assays *HEK293T human embryonic kidney cells* for co-immunoprecipitation assays	NRF2 targets the actin-binding site of BRG1 by mimicking actin's structure to recruit the cRNA polymerase II to the HO-1 promoter but not NQO1	[162,163]
RAC3	C-terminal region of RAC3	Neh4/5	*HeLa human cervical cancer cells* for co-immunoprecipitation and ChIP assays *MCF-7 human breast cancer cells* for RAC3 relavent cancer progression analyses	RAC increases transactivation of NRF2 leading to increased *HMOX1* and *NQO1* gene expressions and cytoprotection against oxidative stress and chemotherapy-induced apoptosis	[164]
SMRT	Receptor interaction domain of SMRT	Neh4/5	*H4IIE rat hepatoma cells* for NRF2/SMRT induction and/or repression assays *HEK293 human embryonic kidney cells* for transfection and reporter assays	SMRT-NRF2 interaction ultimately recruits HDACs to gene promoters causing chromatin condensation that represses the transcription of critical antioxidant genes	[165]
GR	*Unknown*	Neh4/5	*PTEN/KEAP1 mutant mouse*in vivo *models for endogenous PPIs detection Hepa1c1c7 mouse hepatoma cells* for qPCR and ChIP assays *HepG2 human hepatoma cells* for ChIP analysis *HEK293T human embryonic kidney cells* for luciferase reporter assays	GR binds to NRF2 and recruits HDACs, which closes the chromatin and suppresses the expression of key antioxidant genes such as *NQO1* and *GCLM*.	[166]
CHD6	N-terminal region of CHD6	Neh3	*HEK293 human embryonic kidney cells* for transfection and reporter assays *HeLa human cervical cancer cells* for CHD6 siRNA silencing	CHD6 is the essential final step that converts NRF2 from a dormant DNA-binding protein into an active transcription factor, thereby enabling the effective induction of cytoprotective genes	[157]
RXRα/RARα	DNA-binding domain of RXRα/RARα	Neh7	RARα*NRF2*^*−/−*^*and NRF2*^*+/+*^*male mice* for NRF2-dependent antioxidant genes analyses *AREc32 reporter cells* (MCF-7 breast cancer cells stably transfected with an ARE-Luciferase construct) for the identification of retinoic acid induced NRF2 activity RXRα*C5*7BL*/6 male mice* for NRF2 mediated gene expression analyses prove the interaction happens in vivo *HEK293T human embryonic kidney cells* for domain mapping *MCF-7 human breast cancer*, *A549 human non-small cell lung cancer, Caco2 human colon cancer cells* for transfections and luciferase reporter gene activity assays	Activation of RXRα/RARα by retinoic acid causes it to bind NRF2 preventing NRF2 to bind DNA and expression of Phase II detoxification enzymes and this binding likely physically blocks the recruitment of co-activators like CBP/p300	[12,103]

**Table 2:** Other reported NRF2 PTMs and their effects.

PTM	PTM Site on NRF2	Modifier	Ultimate effect	References
Acetylation/Deacetylation	Non defined	CBP/p300 (acetylases) HDAC1, HDAC2, SIRT1, SIRT3 (deacetylases)	- Acetylation by CBP/p300 increases NRF2 stability, promotes its binding to AREs, and enhances transcriptional activity. - Deacetylation by HDACs or SIRT1 generally reduces NRF2's activity by decreasing its DNA-binding affinity and retaining NRF2 in the cytoplasm, thereby facilitating KEAP1-mediated ubiquitination and proteasomal degradation. - In ferroptosis models, SIRT3-mediated deacetylation decreases acetylated NRF2, promotes NRF2 nuclear translocation, increases GPX4 expression, and suppresses ferroptosis.	[154,[167], [168], [169], [170], [171]]
Methylation	Arg437 (Neh1 domain)	PRMT1	Methylation of NRF2 boosts NRF2's transcriptional activity without altering its stability or subcellular localization.	[172]
SUMOylation	Lys110 (nearby Neh4 domain) Lys533 (Neh1 domain)	SUMO1/2 RNF4 Arkadia	SUMOylation can either promote NRF2 degradation or stabilization depending on cellular context: - SUMOylated NRF2 is recognized by RNF4 leading to polyubiquitination and proteasomal degradation. - SUMOylation recruits Arkadia generating atypical K48-linked chains that stabilize NRF2 - Key SUMOylation residues K110 and K533 regulate nuclear import and transactivation - De-SUMOylation at K110 (e.g., K110R; likely SENP1-mediated) protects against myocardial ischemia-reperfusion injury by attenuating ferroptosis. - K110 de-SUMOylation is associated with reduced Tfr expression, lower iron accumulation, and decreased infarct severity.	[[173], [174], [175], [176], [177], [178], [179]]
O-GlcNAcylation	Ser103 (between Neh2/d domains)	OGT	- O-GlcNAcylation by OGT inhibits NRF2's interaction with KEAP1, resulting in NRF2 stabilization, increased nuclear accumulation, and upregulation of antioxidant defense genes - Conversely, under prolonged metabolic stress, excessive O-GlcNAcylation of NRF2 could reduce its nuclear presence and transcriptional efficacy	[[180], [181], [182], [183], [184]]

### 4.1 Other conditional NRF2 partners

Sekine et al. (2016) identified the Mediator complex subunit MED16 as a critical NRF2 cofactor required for robust antioxidant gene expression in response to electrophilic stress [158]. Proteomic analyses revealed MED16 as a direct NRF2-interacting protein, and its disruption markedly reduced the induction of NRF2 target genes following oxidative challenge [158]. The interaction occurs via the Neh4 and Neh5 transactivation domains and is essential for phosphorylation of the RNA Polymerase II C-terminal domain. Loss of MED16 impaired the recruitment of transcriptional machinery and increased sensitivity to oxidative stress, establishing the KEAP1–NRF2–MED16 axis as a major cytoprotective pathway in epithelial and hepatic cells [158].

Zhang et al. (2006) identified BRG1, the ATPase subunit of the SWI/SNF chromatin-remodeling complex, as a selective coactivator of NRF2, promoting the transcription of specific cytoprotective genes, such as *HMOX1* [162]. BRG1 physically associates with NRF2 and is recruited to AREs under oxidative stress, enhancing chromatin accessibility. Interestingly, BRG1 selectively regulates *HMOX1* but not *NQO1*, highlighting that NRF2 coactivator engagement can be gene-specific [162]. In a follow-up study, Zhang et al. (2007) demonstrated that the Neh5 domain of NRF2 mediates its interactions with key coactivators such as CBP and BRG1 [163]. Deletion or mutation of this domain reduced *HMOX1* induction and weakened NRF2–coactivator binding, indicating that Neh5 functions as a condition-specific recruitment center critical for the activation of stress-responsive genes [163].

Kim et al. (2013) showed that the nuclear co-regulator RAC3 (also known as AIB1/SRC-3) acts as a potent NRF2 coactivator [164]. RAC3 interacts directly with the Neh4 and Neh5 domains of NRF2, facilitating its recruitment to AREs and enhancing *HMOX1* expression. This interaction links redox regulation to oncogenic transcriptional control, suggesting that RAC3 integrates oxidative and proliferative signaling networks [164].

In contrast, glucocorticoid receptor (GR) signaling exerts inhibitory control over NRF2 activity. Ki et al. (2005) reported that ligand-activated GR recruits the corepressor SMRT, which binds the Neh4/5 domains and represses NRF2-mediated transcription of detoxifying genes such as GSTA2 [165]. Similarly, Alam et al. (2017) found that GR suppresses histone acetylation and RNA Polymerase II recruitment at ARE-containing promoters [166]. This repression, reversible by histone deacetylase (HDAC) inhibitors, implies that chronic glucocorticoid exposure may dampen NRF2-driven antioxidant defense [166].

Nioi et al. (2005) highlighted the distinct function of the Neh3 domain, which interacts with the chromatin-remodeling factor CHD6 to facilitate transcriptional activation [157]. Loss of this domain significantly impaired the expression of NRF2 target genes without affecting DNA binding, underscoring its role as an independent transactivation module that integrates chromatin-level regulation into NRF2 signaling [157].

Two mechanistically related studies revealed that the nuclear receptors RARα and RXRα negatively regulate NRF2 through direct PPI that impair its transcriptional activity [12,103]. Wang et al. (2007) demonstrated that activation of RARα by all-trans retinoic acid (ATRA) suppresses NRF2 function through direct interaction, thereby reducing its DNA binding to antioxidant response elements (AREs) in MCF-7 and intestinal cells, which were obtained from WT and *NRF2*^−/−^ mice, without altering its nuclear localization [103]. This repression led to the downregulation of canonical NRF2 targets such as *AKR1C1* and *HMOX1*, which was reversible upon RARα antagonism or gene silencing [103]. Subsequently, Wang et al. (2013) identified RXRα, the dimerization partner of RARα, as a novel NRF2 repressor acting through a newly characterized inhibitory region, the Neh7 domain (amino acids 209–316) [12]. RXRα binds NRF2 through its DNA-binding domain and, unlike KEAP1, does not influence the nuclear localization of NRF2 or ARE occupancy [12]. Instead, RXRα blocks the recruitment of transcriptional coactivators such as CBP and RNA Polymerase II, effectively silencing target gene expression. This ligand-independent repression was confirmed across multiple human cell types and in mouse liver and intestine. Notably, RXRα overexpression in A549 lung cancer cells attenuated NRF2 activity and increased chemosensitivity [12]. Together, these findings define two distinct retinoid-mediated repression mechanisms: RARα limits NRF2–DNA binding, whereas RXRα inhibits coactivator recruitment through the Neh7 domain, establishing a molecular link between retinoid signaling and NRF2 inhibition with potential therapeutic implications [12,103].

### 4.2 Other PTMs

#### 4.2.1 NRF2 acetylation

As previously detailed, the interaction between NRF2 and CBP/p300 drives site-specific acetylation, which enhances NRF2 stability, ARE binding, and transcriptional activity. Beyond this CBP/p300-mediated regulation, additional acetylation and deacetylation events further modulate NRF2 function. Proteins like HDACs [167] and sirtuin 1 (SIRT1) [154] function as deacetylating enzymes that generally reduce NRF2 transcriptional activity by decreasing its DNA binding affinity to AREs and promoting its cytoplasmic retention. This facilitates KEAP1-mediated ubiquitination and proteasomal degradation of NRF2, effectively reducing its nuclear activity [154,167,168]. Interestingly, HDAC2 exhibits a divergent role compared to other HDACs. In bronchial epithelial cells HDAC2 deacetylates NRF2, while paradoxically enhancing NRF2 protein stability and its transcriptional activity [167]. SIRT1 deacetylation of specific lysine residues in the model of myocardial ischemia/reperfusion (MI/R) injury can also lead to increased stability and activity of NRF2 [169,170]. Moreover, NRF2 itself directly regulates SIRT1 expression, forming a positive feedback loop that amplifies cytoprotective mechanisms under oxidative stress in MI/R injury [169,170]. In line with this condition-dependent view, Cui et al. (2026) recently demonstrated that SIRT3-mediated deacetylation can also enhance NRF2 activity in sepsis-associated acute kidney injury [171]. In LPS-induced mice and erastin-induced ferroptotic HK-2 cells, reduced SIRT3 expression was associated with increased acetylated NRF2 levels and diminished nuclear NRF2, whereas treatment restored SIRT3 expression, decreased NRF2 acetylation, promoted NRF2 nuclear translocation, and enhanced GPX4 expression, thereby attenuating ferroptosis [171]. These results indicate that the protective effect depends on SIRT3, meaning that when SIRT3 is absent, NRF2 deacetylation and the associated anti-ferroptotic benefit are largely lost.

#### 4.2.2 NRF2 methylation

In addition to acetylation, arginine methylation also modulates NRF2 function. Protein Arginine Methyltransferase 1 (PRMT1) has been identified as a key enzyme that methylates NRF2 at R437 within the Neh1 domain, thereby enhancing its transcriptional activity [172]. PRMT1-mediated methylation facilitates the recruitment of the p300/CBP coactivator complex, leading to increased histone acetylation at ARE promoters, enhanced NRF2 DNA binding, and upregulated transcription of downstream antioxidant genes. This modification strengthens the cellular defense against oxidative stress without affecting NRF2 stability or subcellular localization [172].

Under conditions of elevated oxidative stress, PRMT1-dependent methylation of NRF2 increases, resulting in the induction of specific target genes, including thioredoxin reductase 1 (*TXNRD1*), glutamate-cysteine ligase modifier subunit (*GCLM*), and *HMOX1*, but not *NQO1* [172]. Similarly, SIRT1-mediated deacetylation of NRF2 does not permanently alter *NQO1* expression [154,172], suggesting that *NQO1* regulation may occur independently of NRF2 PTMs.

#### 4.2.3 NRF2 SUMOylation

SUMOylation, one of the most recently characterized PTMs of NRF2, involves the covalent attachment of small ubiquitin-like modifier (SUMO) proteins to lysine residues within specific SUMO-binding motifs [173,185,186]. SUMOylation modulates protein localization, antagonizes ubiquitination, and can either promote or suppress transcriptional activity and protein degradation [[187], [188], [189]].

Early studies on NRF2 SUMOylation revealed that this modification can lead to both degradation and stabilization of NRF2, as well as altered transcriptional activity [173]. NRF2 degradation within the nucleus has been linked to promyelocytic leukemia nuclear bodies (PML-NBs), subnuclear structures involved in stress response and protein turnover. Within these compartments, cross-talk between SUMOylation and ubiquitination occurs via SUMO-targeted ubiquitin ligases (STUbLs), such as RNF4 and Arkadia (RNF111), which recognize polysumoylated proteins through SUMO-interacting motifs (SIMs) and mediate their polyubiquitination and subsequent proteasomal degradation [[190], [191], [192]].

Malloy et al. (2012) provided the first direct evidence linking SUMOylation to the turnover of NRF2 [173]. They showed that NRF2 undergoes SUMO-dependent degradation mediated by RNF4 within PML-NBs. Overexpression of RNF4 reduced NRF2 levels and transcriptional activity, whereas catalytically inactive RNF4 mutants or proteasome inhibition (using MG-132) prevented degradation [173]. NRF2 colocalization with PML and SUMO proteins was confirmed by confocal microscopy analysis, and in vitro assays demonstrated that NRF2 is directly modified by both SUMO-1 and SUMO-2. These findings established SUMOylation as a nuclear regulatory mechanism that promotes NRF2 degradation via RNF4-mediated proteasomal targeting [173].

More recent evidence suggests that SUMOylation can also stabilize NRF2 [174,175]. McIntosh et al. (2018) reported that, after nuclear translocation, NRF2 undergoes poly-SUMOylation, which enables K48-linked ubiquitination by Arkadia, interestingly, resulting in NRF2 stabilization rather than degradation [174]. Co-expression of Arkadia with wild-type ubiquitin enhanced ARE-driven transcription, while mutation of K48 ubiquitin chains reduced *HMOX1* promoter activity. Although K48-linked ubiquitination typically marks proteins for degradation, in this aspect, it stabilizes NRF2 and promotes transcriptional activity, possibly by delaying RNF4-mediated turnover during oxidative stress [174].

In addition to these mechanistic evidences, specific SUMOylation sites on NRF2 have been identified. Guo et al. (2019) demonstrated that lysine 110 (K110) is the conserved SUMO1 binding site on NRF2 and described a novel signaling pathway that promotes de novo serine synthesis through NRF2 SUMOylation, maintaining HCC tumorigenesis [176]. SUMO-1 modification was also found to be essential for NRF2–MafG complex formation [177]. Later, Walters et al. (2021) identified K110 (Neh4) and K533 (Neh1) as SUMO-2 acceptor sites required for nuclear translocation, stabilization, and transactivation of NRF2 under oxidative stress conditions (e.g., arsenic trioxide exposure) [175]. Loss of these lysine residues impaired NRF2 transcriptional activity and stability. Similarly, He et al. (2018) demonstrated that the loss of the SUMO-conjugating enzyme Ubc9 in mice enhanced NRF2 degradation, resulting in severe diabetes due to oxidative stress–induced β-cell dysfunction [178]. Extending the functional relevance of the K110 site, Shi et al. (2026) demonstrated that de-SUMOylation of NRF2 at K110 exerts a protective effect in myocardial ischemia-reperfusion injury (MIRI). By using NRF2 K110R knock-in mice, which mimic de-SUMOylated NRF2, it was observed no marked abnormalities in cardiac morphology or function under basal conditions, but found that NRF2 de-SUMOylation significantly alleviated myocardial injury under ischemia-reperfusion stress [179]. Mechanistically, this protective effect was associated with reduced ferroptosis and decreased transferrin receptor (Tfr) expression, while pharmacological inhibition of ferroptosis diminished the difference between wild-type and K110R mice [179]. These findings further support the idea that modification of NRF2 at K110 acts as a condition dependent regulatory switch and indicate that reversal of SUMOylation can also shape NRF2-dependent stress responses in a tissue- and disease-specific manner.

Collectively, these findings suggest that SUMOylation exerts a dual control over NRF2, functioning as a cell- or condition-specific switch between NRF2 stabilization and degradation. Further studies are required to clarify how SUMO modifications interact with other PTMs and regulatory pathways to precisely regulate NRF2 signaling.

#### 4.2.4 NRF2 O-GlcNAcylation

O-linked β-N-acetylglucosaminylation (O-GlcNAcylation) represents a reversible PTM where N-acetylglucosamine (GlcNAc) groups attach to the hydroxyl groups of serine and threonine residues across nuclear and cytoplasmic, and mitochondrial proteins [193,194]. O-GlcNAcylation influences both the stability and activity levels, as well as the subcellular distribution, protein-protein binding capabilities, and phosphorylation states of the target protein. Unlike the glycosylation process that produces complex glycan structures on secreted or membrane-bound proteins, O-GlcNAcylation occurs as an intracellular modification without further elongation. This process is catalyzed by O-GlcNAc transferase (OGT) enzyme by using uridine diphosphate N-acetylglucosamine (UDP-GlcNAc) as its donor substrate, and is reversed by O-GlcNAcase (OGA) enzyme, which hydrolyzes the glycosidic bond [195,196].

The donor substrate, UDP-GlcNAc, is the end product of the hexosamine biosynthetic pathway (HBP). Almost 2–5% of glucose entering the cell is directed to the HBP to convert it into UDP-GlcNAc through the process that combines glucose with inputs from glutamine, acetyl-CoA, and uridineZeidan [197]. Therefore, the metabolic changes in nutrients and hormonal signals cause cellular O-GlcNAcylation levels to adjust their activity as a metabolic sensor.

O-GlcNAcylation functions as a vital regulatory mechanism for oxidative stress through its direct modification of NRF2/KEAP1 components. Through this function, it establishes a relationship between nutrient sensing and stress response mechanisms. The first direct demonstration that NRF2 can be modified by O-GlcNAcylation was reported by Dieter et al. (2015), who investigated the mechanism from a cardiac perspective [180]. Using O-GlcNAc-enhanced cardiomyocytes (H9C2 cell line), they demonstrated that the NRF2 protein is O-GlcNAc-modified, and this modification also upregulates the expression of canonical antioxidant genes, including catalase, *GCLM*, and *HMOX1* [180]. Interestingly, in OGT knockout mice, while the NRF2 protein increased with OGT loss, its transcriptional activity decreased, suggesting that O-GlcNAcylation might be essential for NRF2 stability and may also be important in transcriptional activity [180].

Later, Chen et al. (2017) reported that KEAP1 is also a direct substrate of OGT, and O-GlcNAcylation of KEAP1 at S104 residue enhances its E3 ligase function to ubiquitinate and degrade NRF2 under nutrient-rich conditions [181]. However, during nutrient deprivation or metabolic stress, KEAP1 glycosylation is reduced, resulting in NRF2 stabilization and the activation of stress response genes [181]. This study did not directly assess NRF2 glycosylation; however, it established a nutrient-sensitive upstream checkpoint regulating NRF2 turnover, thereby providing a mechanistic link between metabolic state and redox homeostasis [181]. These findings offer a compelling explanation for the paradoxical in vivo observation by Dieter et al. (2015) that NRF2 protein levels increased despite OGT deletion [180]. This suggests that the loss of KEAP1 O-GlcNAcylation reduces its E3 ligase activity, which allows for NRF2 accumulation. However, the absence of direct NRF2 glycosylation may impair its transcriptional function.

In contrast, Costa et al. (2023) reported that in diabetic rats, hyperglycemia led to elevated O-GlcNAc-modified NRF2, which surprisingly decreased nuclear localization and transcriptional activity of NRF2, contributing to oxidative kidney damage [182]. Increased O-GlcNAc levels, induced by pharmacological inhibition of OGA, in HEK293 cells decreased NRF2 activity, while pharmacological activation of NRF2 rescued antioxidant gene expression. These observations may imply a limiting effect, where excessive O-GlcNAcylation under prolonged metabolic stress shifts from being activating to being inhibitory [182].

Dai et al. (2020) demonstrated that in hypopharyngeal squamous cell carcinoma (HSCC), increased O-GlcNAcylation results in the stabilization of NRF2 through the PI3K/AKT signaling pathway [183]. Knockdown of O-GlcNAcylation reduced NRF2 levels and impaired cell growth, while forced NRF2 expression restored malignant behaviors [183]. This suggested that O-GlcNAcylation may represent a pro-tumorigenic signal by promoting NRF2 stabilization in cancers, contrasting with the inhibitory effect seen in hyperglycemia.

Recently, Zhang et al. (2024) showed that S103 is a direct O-GlcNAcylation site on NRF2 [184]. This modification blocks KEAP1 binding, thereby promoting NRF2 stability, nuclear accumulation, and transcriptional activity. In both cellular and xenograft lung cancer models, O-GlcNAcylated NRF2 at S103 contributed to tumor aggressiveness and cisplatin resistance [184]. Oxidative stress and cisplatin treatment increased AMPK-driven phosphorylation of OGT at T444, which increased its binding to NRF2 and enhanced O-GlcNAcylation under redox disturbances. This study provides molecular evidence that O-GlcNAcylation stabilizes NRF2 and is a direct substrate with known condition-specific outcomes [184].

Taken together, these findings underscore a critical role for O-GlcNAcylation and place this PTM at the crossroad where nutrient sensing meets oxidative stress signaling. In the future, it may be beneficial to explore therapeutic strategies that modulate KEAP1 and NRF2 O-GlcNAcylation, thereby enabling regulation of the NRF2 response under metabolic conditions.

## 5 Conclusions and future perspective

Recent system biology approaches have demonstrated that PTMs influence cellular processes not only through local structural changes but also by altering the global positioning of proteins within protein interaction networks. Proteins carrying specific PTMs such as phosphorylation, SUMOylation, and acetylation tend to occupy highly characteristic networks underscoring a broader principle that PTMs frequently operate by triggering distinct PTM-dependent PPIs, often mediated by specialized “reader” proteins that recognize the modified residue, while also indirectly reshaping PPIs by altering protein conformation or subcellular localization [198,199].

Along with this, the reverse relationship is equally important and increasingly recognized. PPIs themselves can remodel or dictate specific PTM patterns by exposing or shielding regulatory motifs, recruiting modifying enzymes, or stabilizing conformational states that favor or prevent particular PTMs [200]. In this bidirectional model, partner proteins function not merely as random downstream effectors but as active sculptors of a protein's PTM code, redefining its modification, topology, and regulatory fate.

Despite its relevance, this systems biology perspective has not yet been applied to NRF2, even though this protein is heavily modified (43 documented PTMs) and engages in a remarkably diverse interactome spanning KEAP1, β-TrCP, PIN1, RXRα, chromatin remodelers, metabolic sensors, and nuclear co-regulators. Understanding whether particular NRF2 PTMs position the protein at more central or peripheral network nodes, and, conversely, whether distinct NRF2-binding partners reshape its PTM accessibility through sequestration, competition, or conformational selection, could open up a new organizational reasoning underpinning NRF2 signaling. Integrating a global PTM–protein interaction network with NRF2-specific datasets may represent a transformative opportunity to decode how different stress, metabolic, and tissue-specific conditions reprogram NRF2 activity through a dynamic, mutually reinforcing PTM–PPI network.

Yet, building such an integrated PTM–PPI network for NRF2 is challenging because the NRF2 literature is not only incomplete but also marked by longstanding debates, conflicting evidence, and unresolved interpretations. The ongoing discussion on the functional relevance of S40 phosphorylation exemplifies how incomplete or conflicting data can persist for decades without resolution. Although NRF2 S40 phosphorylation was initially proposed to be essential for KEAP1 release and NRF2 activation, multiple studies now dispute this model. S40A mutants respond normally to electrophilic activators [55]; structural predictions indicate that the KEAP1–Neh2 complex remains intact even when S40 is phosphorylated (Fig. 3A) and evolutionary analyses reveal that S40 is poorly conserved. Additionally, concerns about the specificity of commonly used pS40 antibodies raise doubts about past observations. Overall, S40 appears to be a genuine phosphosite, but its functional relevance to NRF2 activation remains unresolved.

Similarly, the phosphorylation-dependent interaction between NRF2 and PIN1 represents an emerging but still mechanistically unresolved axis, with phosphorylation-dependent binding, potential competition with KEAP1, and condition-specific positive or negative regulation, all contributing to a complex and sometimes contradictory picture. Clarifying this regulatory mechanism will require studies that go beyond phospho-peptide mimics to examine domain-based NRF2-PIN1 interaction, determine whether PIN1 catalyzes proline isomerisation, and define how KEAP1 phosphorylation alters the broader NRF2 interactome. Additionally, describing the conditions under which PIN1 stabilizes versus suppresses NRF2 activity across different tissues, stress conditions, and oncogenic microenvironments will be essential for understanding whether PIN1 acts as a selective activator, an indirect repressor, or a condition-dependent modulator of the NRF2/KEAP1 axis. Resolving these uncertainties could reveal that PIN1 is a critical adjustable switch within the NRF2 regulatory network, making it a promising therapeutic target.

Growing evidence indicates that nuclear regulation of NRF2 is more intricate than previously known, with WDR23 and CBP/p300 forming two mechanistically distinct control routes. WDR23, through its cytoplasmic and nuclear isoforms, appears to mediate compartment-specific degradation of NRF2; however, the molecular mechanisms underlying isoform switching and crosstalk with the KEAP1/CUL3 system remain unresolved. In parallel, CBP/p300 modulates NRF2 activity through a direct engagement of its intrinsically disordered Neh4 and Neh5 domains via its TAZ1/TAZ2 domains, as well as acetylation of critical lysine clusters within Neh1 and Neh3 domains. These interactions not only enhance NRF2 chromatin binding and transcriptional output but also stabilize NRF2 by disrupting KEAP1 binding. But, how acetylation across different NRF2 domains is coordinated, how CBP/p300 prioritizes NRF2 over other competing factors (HIF-1α, CITED2, and STAT1), and how nuclear degradation via WDR23 crosstalk with acetylation-driven stabilization remain largely unknown. Future studies combining structural analyses of disordered-region folding, acetylation-site mapping, isoform-specific interactomics, and nuclear turnover kinetics will be essential to define how these regulatory axes collectively influence the nuclear lifetime of NRF2.

In addition, most of the NRF2-interacting partners involved in transcriptional control, including MED16, BRG1, RAC3/SRC-3, CHD6, RARα, and RXRα, function primarily within the nucleus and therefore represent key components of NRF2's nuclear regulatory network. However, despite their clear functional relevance, these interactions remain incompletely characterized and can be reasonably considered “unresolved PPIs”. For most of them, their PTM dependence is unknown, and binding interfaces have not been fully mapped. Moreover, several of these interactions occur through intrinsically disordered regions of NRF2, making them more challenging to resolve. Several PTMs such as SUMOylation and O-GlcNAcylation also remain underexplored in terms of their PPIs despite indications that they function as metabolic or condition-specific switches influencing NRF2 stability.

Advanced systems biology platforms such as Cytoscape-based PTMOracle [201], iPTMnet [202] and BioBERT [203] represent how PTMs and PPIs can be co-visualized and co-analyzed within a network framework. These tools allow researchers to overlay PTM data onto known PPI networks, detect potential binding interfaces, and highlight nodes where PTM–PPI crosstalk may drive regulatory behaviour, thereby providing a useful starting point for hypothesis generation in NRF2 research. Although their current coverage of NRF2-specific PTM–PPI relationships remains limited, further development of these platforms together with the implementation of machine-learning strategies such as structure-based prediction of PTM-sensitive regions, and large-scale text mining of PTM–PPI associations, is expected to make it feasible to identify which modifications are most likely to modulate NRF2's interaction network and, conversely, how network rewiring may reshape its modification code. Systematic integration of such next generation bioinformatic tools into NRF2 research will be essential for dissecting its complex regulatory architecture and is likely to accelerate the discovery of novel regulatory nodes and therapeutic targets.

Finally, the KEAP1–NRF2 axis is a significant therapeutic target across a wide range of pathologies, including neurodegeneration, chronic inflammation, metabolic disease, and cancer. KEAP1-targeting NRF2 activators have already entered clinical practice, with dimethyl fumarate (BG-12, Tecfidera®) approved for relapsing–remitting multiple sclerosis and psoriasis, and RTA-408 (omaveloxolone, SKYCLARYS®) becoming the first FDA-approved therapy for Friedreich ataxia [204]. In contrast to these broadly acting electrophiles, the small molecule PHAR offers a more selective strategy that disrupts the β-TrCP–NRF2 phosphodegron interaction, induces mild, KEAP1-independent NRF2 activation, and shows anti-inflammatory and anti-fibrotic efficacy in preclinical models of liver disease, but has not yet advanced to human trials [75]. Parallel work on molecular glues that stabilize E3 ligase–substrate complexes suggests an opposite β-TrCP-centred approach for NRF2-addicted cancers, in which enforced β-TrCP–NRF2 proximity could drive selective NRF2 degradation [205,206]. However, core NRF2 regulators such as KEAP1, β-TrCP, and PIN1 each participate in numerous additional signaling pathways, so non-specific activation or inhibition of these proteins risks broad pleiotropic effects. We therefore propose that future therapeutic efforts should increasingly shift from targeting single molecules to modulating specific PPIs, and ultimately PTM-defined subnetworks within the NRF2 axis, an approach that holds greater promise for achieving condition-selective NRF2 modulation with an improved balance between efficacy and safety.

## CRediT authorship contribution statement

**Adem Ozleyen:** Visualization, Writing – original draft. **Seda Savranoglu Kulabas:** Visualization, Writing – original draft. **Miroslav Novak:** Data curation, Visualization, Writing – original draft. **Milena Cichoń:** Writing – original draft. **Cristina Matas De Las Heras:** Writing – original draft. **Tadashi Honda:** Data curation. **Richard G. Doveston:** Writing – original draft, Writing – review & editing. **Albena T. Dinkova-Kostova:** Writing – original draft, Writing – review & editing. **Anna Grochot-Przeczek:** Funding acquisition, Writing – original draft, Writing – review & editing. **Tugba Boyunegmez Tumer:** Conceptualization, Funding acquisition, Supervision, Visualization, Writing – original draft, Writing – review & editing.

## Declaration of competing interest

The authors declare that they have no known competing financial interests or personal relationships that could have appeared to influence the work reported in this paper.

## Data Availability

Data will be made available on request.
