# Supplementary figures and images for: Interplay between NRF2 post-translational modifications and protein-protein interactions: Perspectives from emerging structural and functional evidence
Source: Arch Biochem Biophys. 2026 Aug;782:None. doi: 10.1016/j.abb.2026.110847 (PMC13271694; doi:10.1016/j.abb.2026.110847)

# Figure 4A

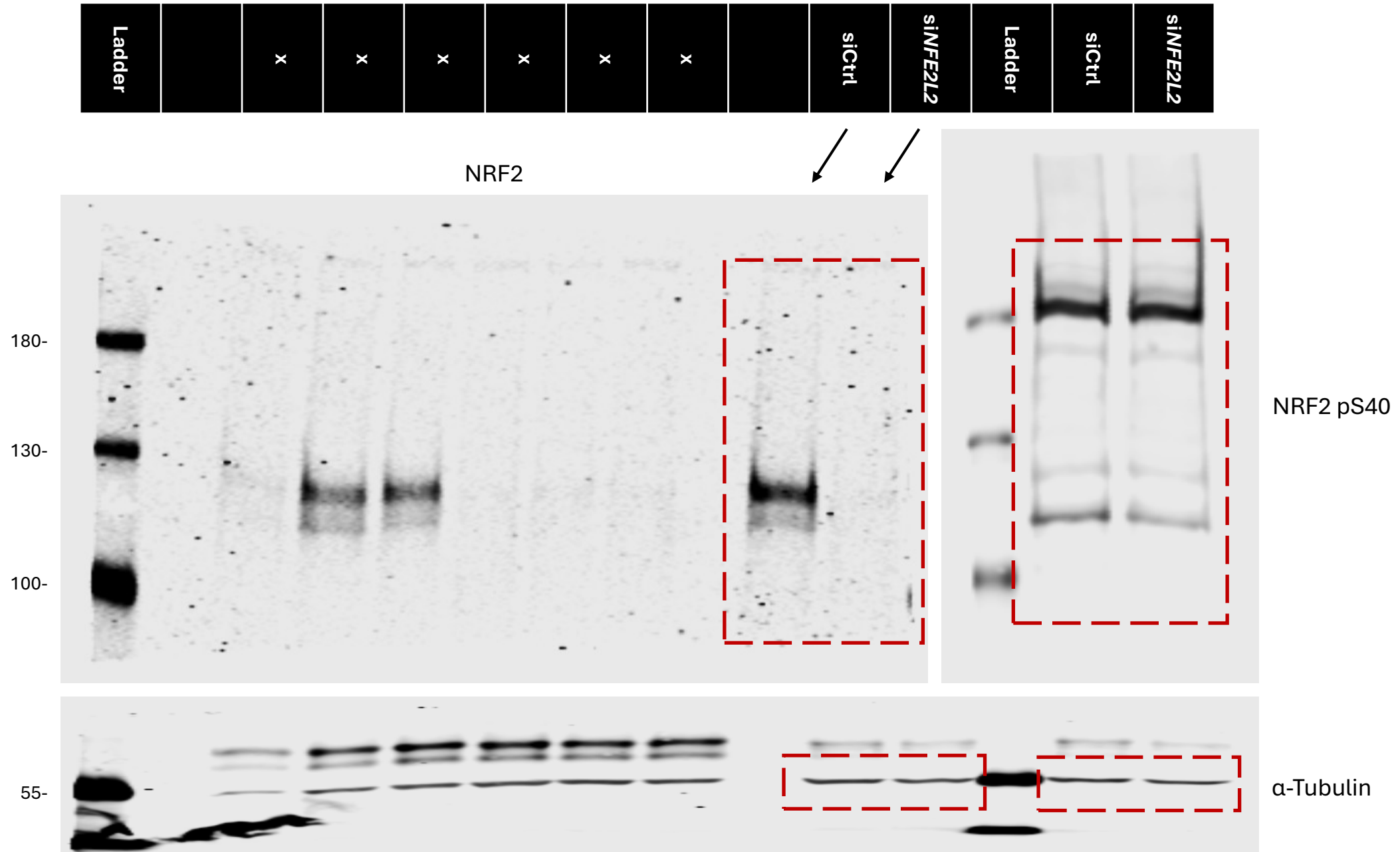

Figure 4B Top

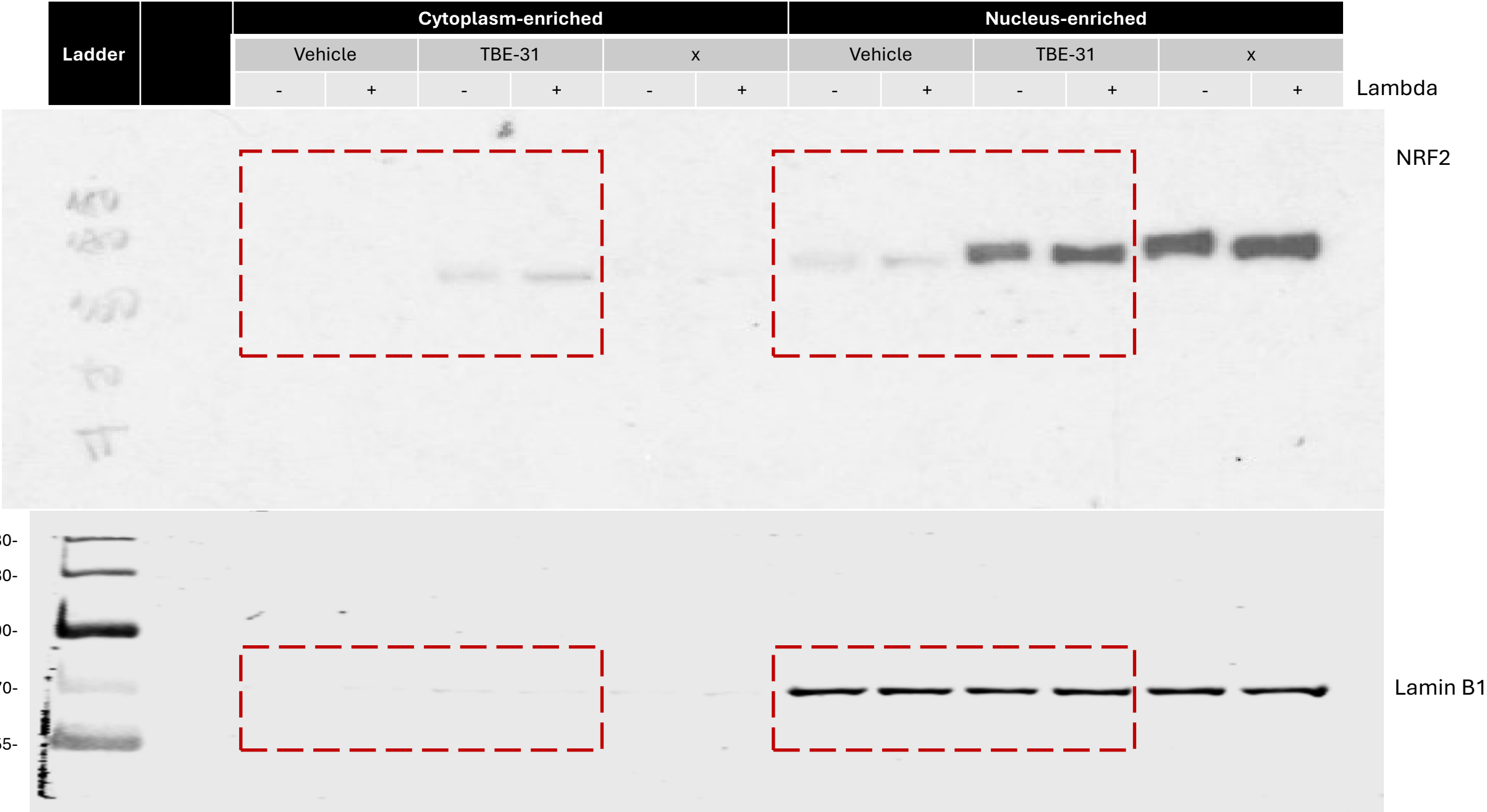

# Figure 4B Top

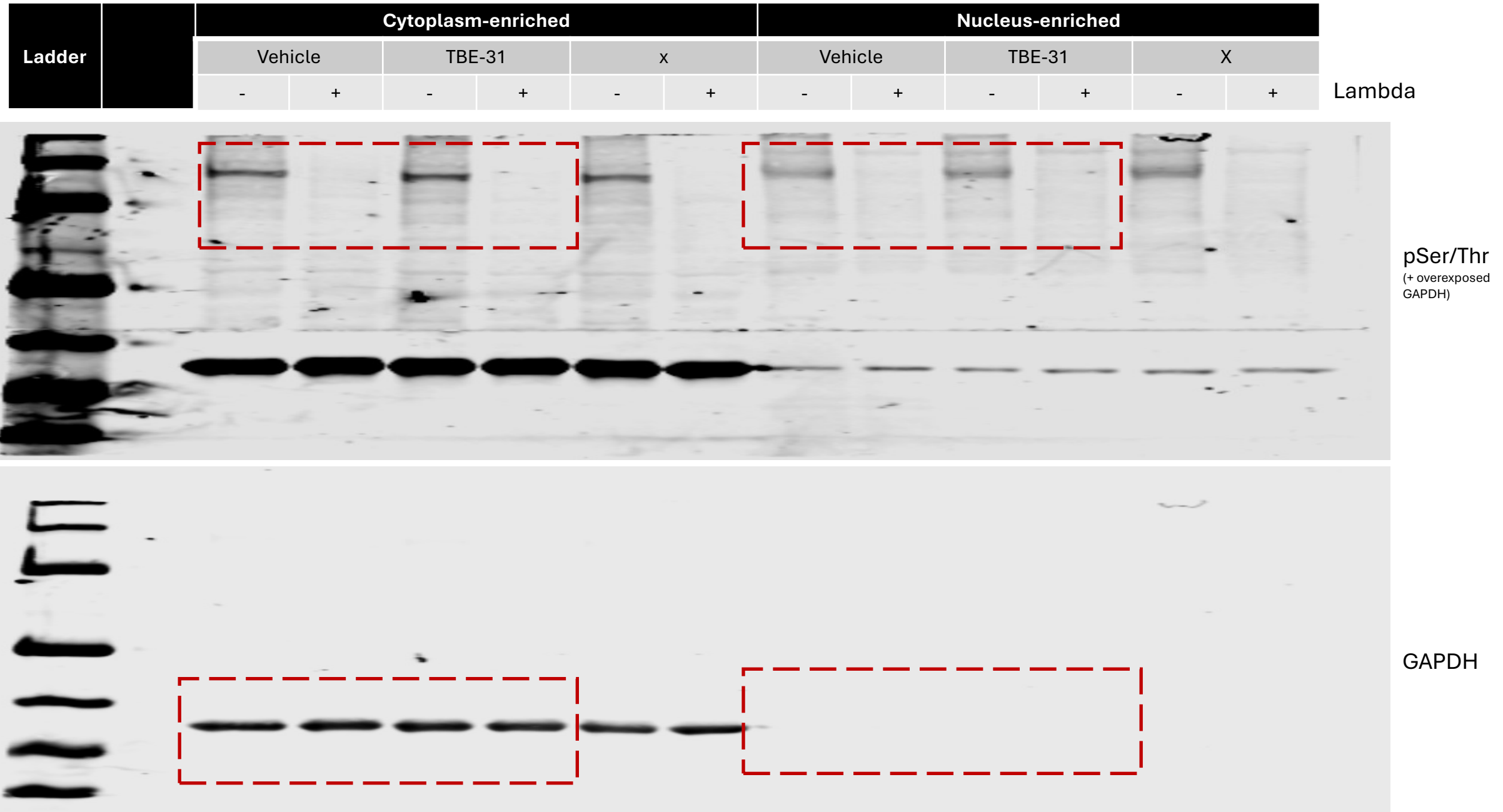

# Figure 4B Bottom

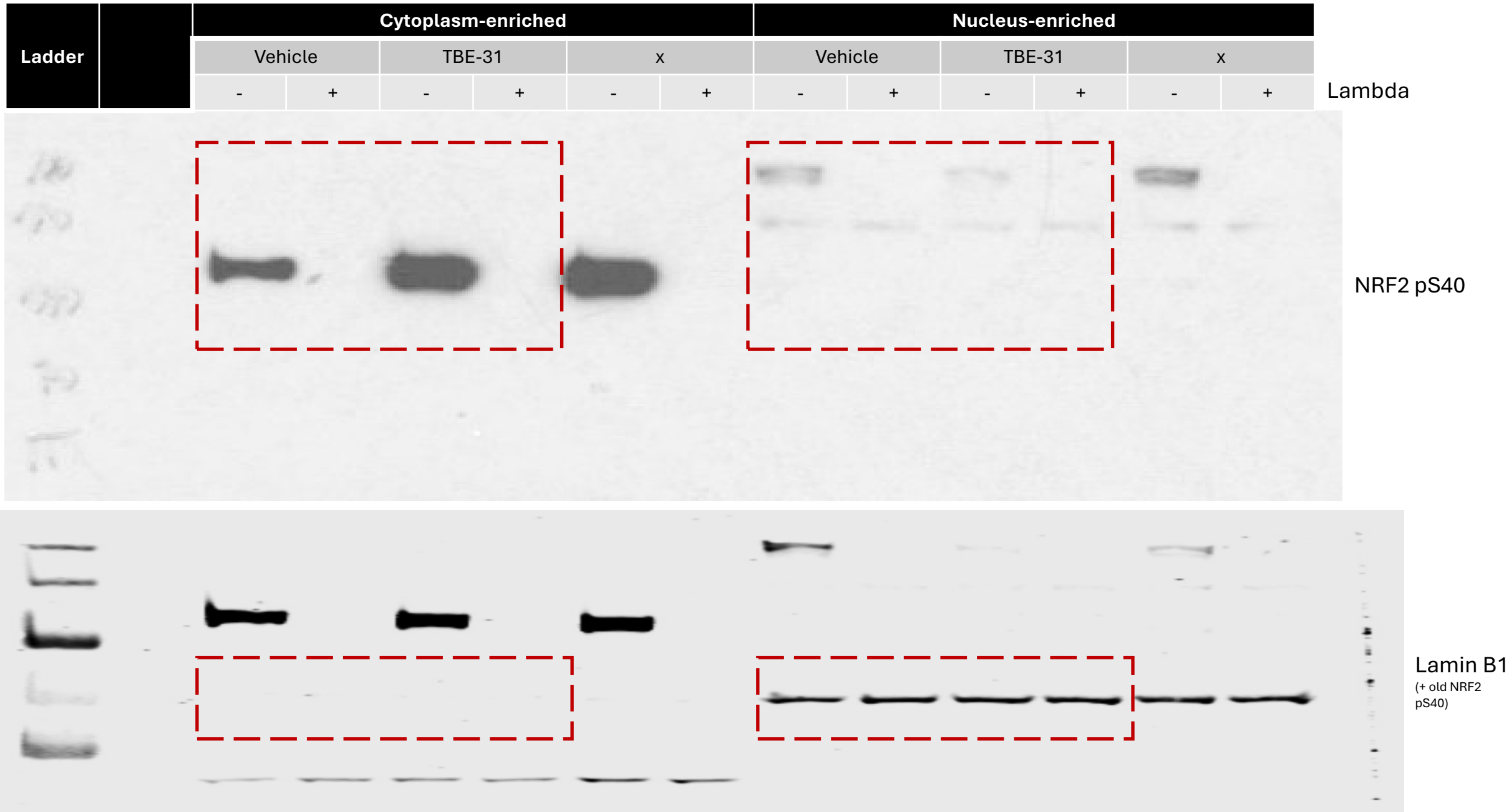

Figure 4B Bottom

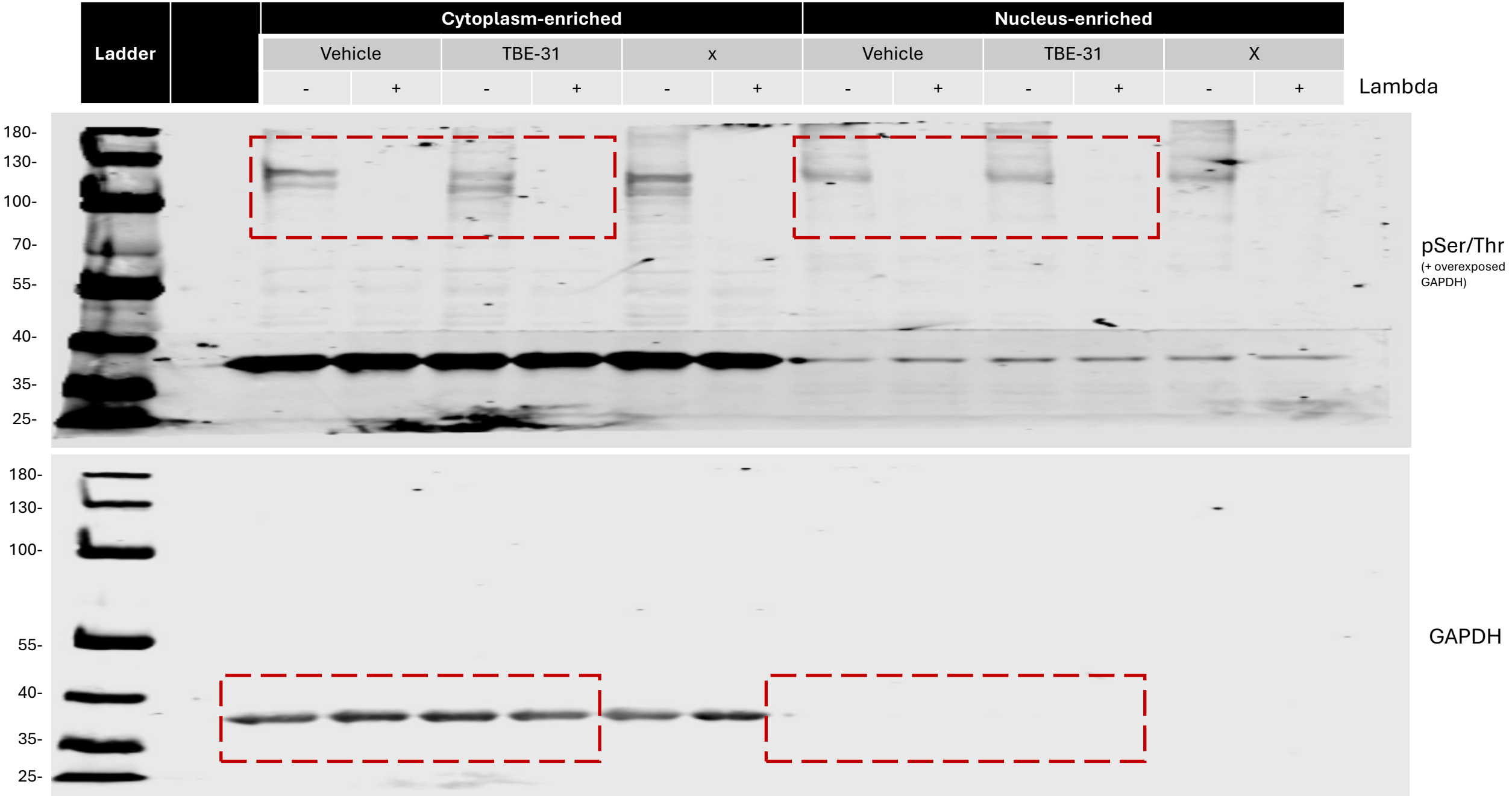

Supplement: Multimedia component 1 [file mmc1.pdf]
